# Supplementary material for: Type I Interferons Drive the Gastrointestinal Inflammatory Response in a Mouse Model of Parkinson’s Disease
Source: Gastro Hep Adv. 2026 Mar 19;5(6):100929. doi: 10.1016/j.gastha.2026.100929 (PMC13098579; doi:10.1016/j.gastha.2026.100929)
Supplement: Supplementary Material [file mmc1.pdf]

Supplementary Methods

Cardiac puncture, plasma extraction, and tissue collection

WT and IFNAR1$^{-/-}$ mice were anesthetized via an i.p injection of Ketamine (100mg/kg) (ilium Ketamil) + Xylazine (10mg/kg) (ilium Xylazil-20). Blood was removed via cardiac puncture and plasma extracted. The colon was removed then the intestine was excised and separated into duodenum, jejunum and ileum with all tissues snap frozen in liquid nitrogen and stored at -80°C.

RNA isolation and cDNA synthesis

RNA was isolated from intestinal tissues (approximately 50-100mg) using TRIzol (Invitrogen, 15596018). Organoid samples were harvested using Corning® Cell Recovery Solution (Corning®, 354253) and pelleted before using RNAeasy mini kit (Qiagen, 74104) as per manufacturer's instructions. RNA samples were DNAse treated with an Ambion TURBO DNA-free™ Kit (Life Technologies), as per manufacturers guidelines. RNA was reverse transcribed into cDNA using a High-capacity RNA-to-cDNA Reverse Transcription Kit (4368814, Applied Biosystems)

Real time quantitative polymerase chain reaction (RT-qPCR)

All qPCR was performed in triplicate in standard 384-well plates (4309849, Applied Biosciences, Scoresby, VIC, Australia), with real time quantitative gene expression determined using Taqman probles (**Supp Table 2**) and analysed by the Quant Studio 6 Flex Real-Time PCR System (Invitrogen).

Western blot analysis

Western blot analysis was performed on 50µg of protein. Membranes were incubated with the primary antibodies **(Supp Table 3)** for 24 hours at 4°C and secondary antibodies for 1.5 hours at RT. Signals detected using an ECL Prime Detection kit (GE Healthcare Life Sciences) and visualised with a ChemiDoc™ imaging XRS+ system (Bio-Rad).

Enzyme Linked ImmunoSorbent Assay (ELISA)

A Mouse TNF-alpha Duoset ELISA (R&D Systems, DY410) was used to analyse TNFα levels in mouse plasma and in protein samples extracted from WT and IFNAR1$^{-/-}$ mice gut tissues.

Supplementary Tables

Cell Type	Antibody	Supplier	Primary dilution	Secondary Antibody
**All**	Rabbit Anti-alpha-synuclein	Cell Signalling (#4179)	1:200	Alexa Fluor® 488 Goat anti-mouse (A-11001)
**All**	Rabbit Phospho Anti-alpha-synuclein	Cell Signalling (#23706)	1:200	Alexa Fluor® 594 Goat anti-rabbit (A-21207)
**Organoids**	Purified Mouse Anti-E-cadherin monoclonal	BD Transduction Laboratories ™ (#610181)	1:300	Alexa Fluor® 594 Goat anti-rabbit (A-21207)
**Organoids**	Rabbit Lysozyme/Muramidase polyclonal	Thermo Scientific (RB-372)	1:200	Alexa Fluor® 488 Goat anti-mouse (A-11001)
**Organoids**	Mouse Chromogranin A monoclonal	Santa Cruz (#393941)	1:250	Alexa Fluor® 594 Goat anti-rabbit (A-21207)
**Whole gut IHC**	Mouse GFAP monoclonal	Cell Signalling (#3670)	1:1000	Alexa Fluor® 488 Goat anti-mouse (A-11001)

Table 1. Antibodies for immunofluorescence analysis

Gene	Species	Inventory #	Amplicon length (bp)
B2M	Mouse	Mm00437762_m1	77
IFNβ	Mouse	Mm00439552_s1	69
IRF7	Mouse	Mm00516788_m1	67
IL-1β	Mouse	Mm01336189_m1	63
TNFα	Mouse	Mm00443258_m1	81
IL-6	Mouse	Mm00446190_m1	78

Table 2. TaqMan probes used for qPCR analysis

Antibody	Type	Supplier	Catalogue #	Dilution	Species
**Anti-STAT1**	Primary	Cell Signalling	9172	1:1000	**Rabbit**
**Anti-Phospho-STAT1**	Primary	Cell Signalling	9167	1:1000	**Rabbit**
**Anti-NFκB p65**	Primary	Cell Signalling	8242	1:1000	**Rabbit**
**Anti-Phospho-NFκB p65**	Primary	Cell Signalling	3031	1:1000	**Rabbit**
**Anti-β-Actin**	Primary	Sigma-Aldrich	A5441	1:1000	**Mouse**
**Anti-GFAP**	Primary	Cell Signalling	3670S	1:1000	**Rabbit**
**Anti-IRF3**	Primary	Cell Signalling	4302S	1:1000	**Rabbit**
**Anti-STAT3**	Primary	Cell Signalling	4904	1:1000	**Rabbit**
**Anti-Phospho-STAT3**	Primary	Cell Signalling	9145S	1:1000	**Rabbit**
**Anti-IFNβ**	Primary	Santa Cruz	57201	1:500	**Rat**
**Anti-P-S129A αSynuclein**	Primary	Cell Signalling	23706	1:1000	**Rabbit**
**Anti-Total αSynuclein**	Primary	Cell Signalling	4179	1:1000	**Rabbit**
**Goat Anti-Rabbit Immunoglobulins/HRP**	Secondary	Dako	P0448	1:1000	**Goat**
**Goat Anti-Mouse Immunoglobulins/HRP**	Secondary	Dako	P0447	1:1000	**Goat**
**Goat Anti-Rat Immunoglobulins/HRP**	Secondary	Abcam	97057	1:1000	**Goat**

Table 3. Western blot analysis antibodies

Supplementary figures

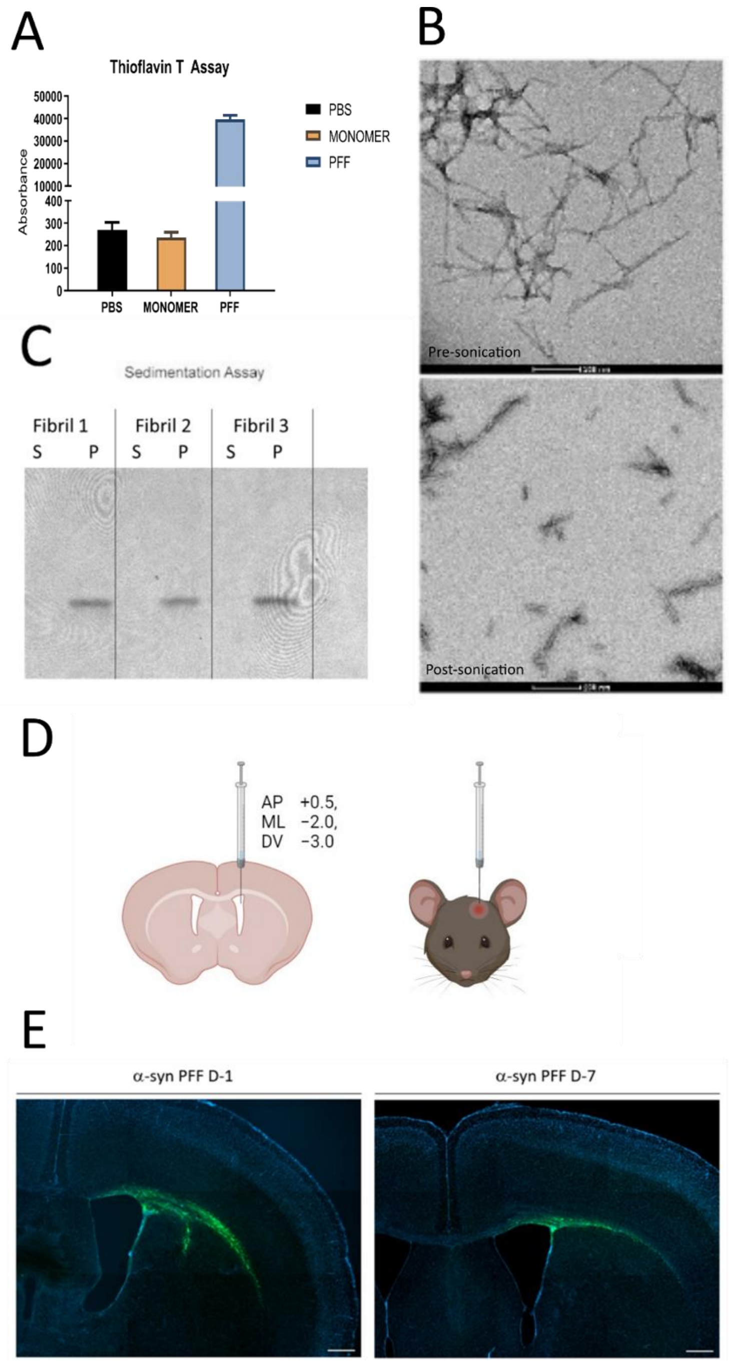

Supplementary Figure 1. Validation of the alpha synuclein PFF intrastriatal injection model

A, Thioflavin T assay confirming increased β-sheet structures in a-Synuclein PFF, when compared to both monomer and vehicle control. **B,** TEM images of pre- (i) and post- (ii) sonicated preparations of α-Syn PFFs showing decreased length (approximately 50nm) of fibrils. **C,** Sedimentation assay confirming the presence of α-Syn PFFs in the pelleted Representative images of 30µm cryosections of mouse brain at 1- and 7-days post injection with 488-labelled α-Syn PFFs. Co-stained with DAPI. Scale bar =100µm.

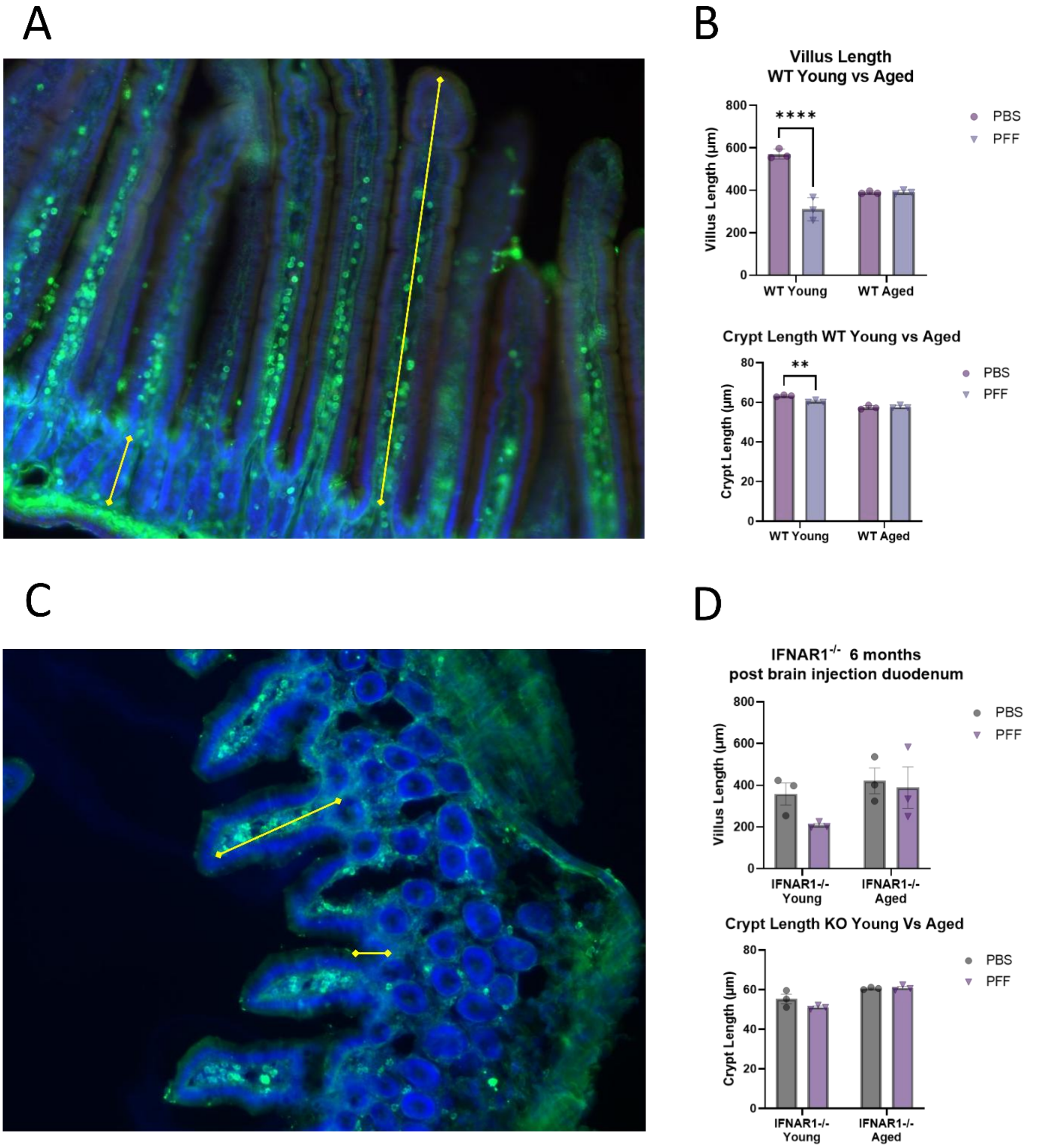

Supplementary Figure 2. Crypt and villus length is altered in young wildtype, but not IFNAR1$^{-/-}$ mice, following an intrastriatal injection of a-Syn PFFs (6-months p.i).

Young and aged WT **(A, B)** and IFNAR1$^{-/-}$ **(C, D)** mice duodenal tissue was analysed for villus and crypt length at 6 months post-injection of a-Syn PFFs or vehicle into the striatum. Representative images of 30μm cryosections stained with DAPI (blue) and Anti-GFAP (green) with 3 villus/crypt per cryosection counted, 10 cryosections per mouse, and 3 mice analysed. Data analysed with Two-way ANOVA, Tukey's Multiple Comparison's, data expressed as mean±SEM, n=3, **p ≤ 0.01, ****p≤ 0.0001.

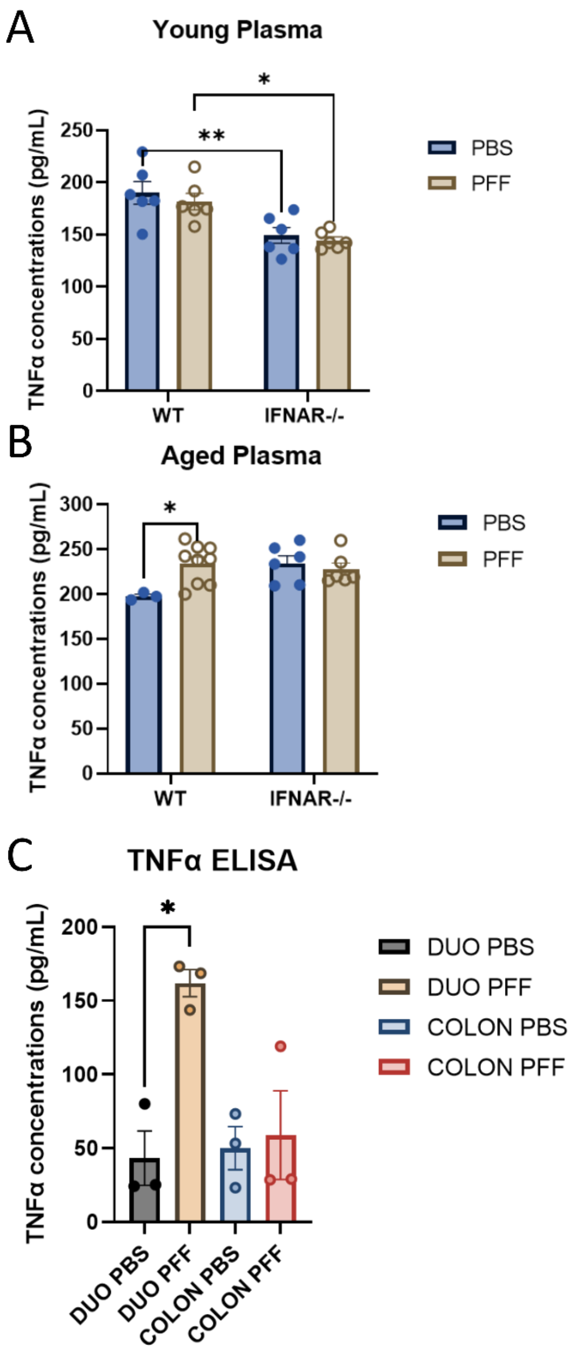

Supplementary Figure 3. TNFα levels are elevated in the plasma and duodenum of aged wildtype mice, but not IFNAR1$^{-/-}$ mice, following an intrastriatal injection of a-Syn PFFs (6-months p.i.)

Young (10-12 weeks of age) or Aged (40-50 weeks of age) wildtype and IFNAR1$^{-/-}$ animals were injected with a-Syn PFFs or vehicle and levels of TNFα in plasma (A, B) or duodenum and colon (C) determined by ELISA at 6-months post injection. Data expressed as mean±SEM, two-way ANOVA, Tukey's multiple comparisons test, * = p≤0.05, ** = p≤0.01.

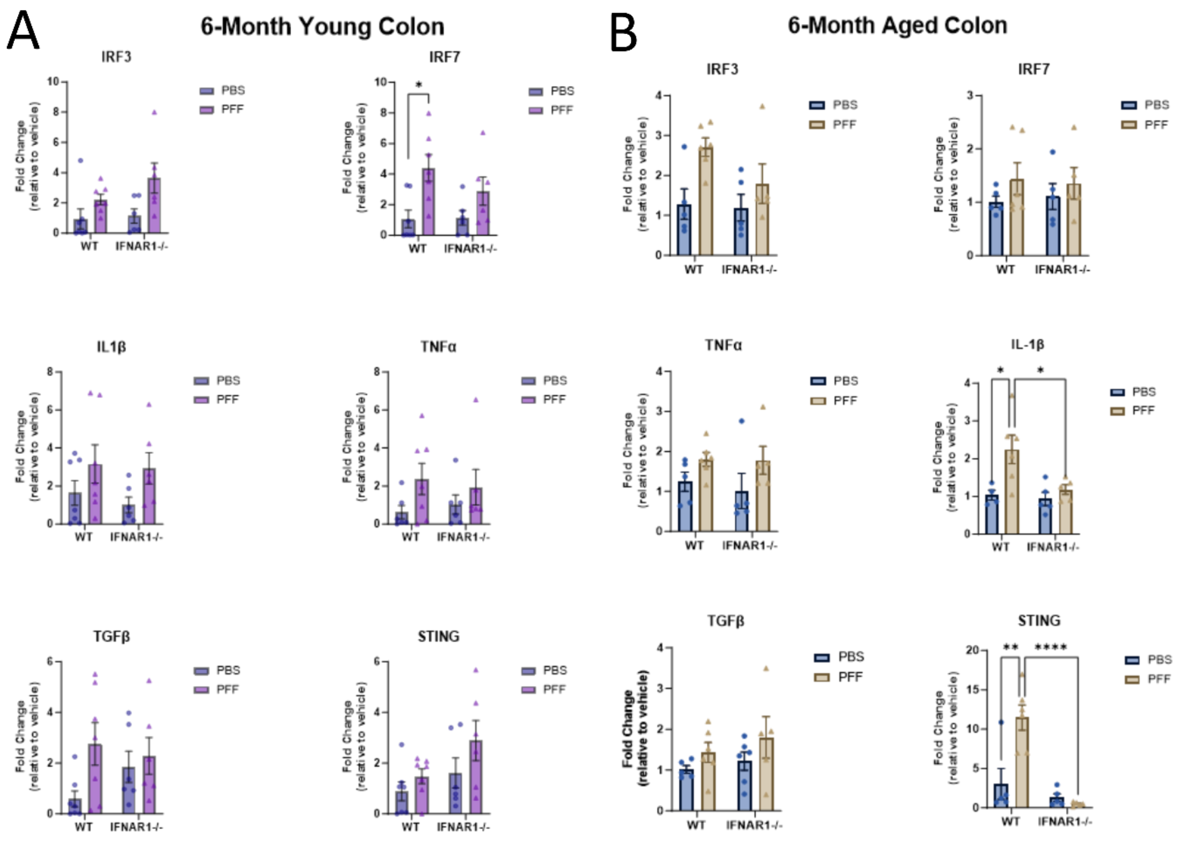

Supplementary Figure 4. IL1β and STING expression are upregulated in the colon of aged wildtype, but not IFNAR1$^{-/-}$ mice following an intrastriatal injection of a-Syn PFFs (6-months p.i.)

Colons from young (10-12 weeks of age when injected) **(A)** and aged (40-50 weeks of age when injected) **(B)** WT and IFNAR1$^{-/-}$ mice were analysed at 6-months after receiving an intrastriatal injection of α-Syn PFFs (8μg) or vehicle. mRNA expression of Interferon regulatory factor 3 & 7 (IRF3/7), Tumour Necrosis Factor Alpha (TNFα), Interleukin 1-beta (IL-1β), Transforming Growth Factor Beta (TGFβ), and Stimulator of Interferon Genes (STING) was determined by qPCR analysis. Data expressed relative to the housekeeping gene B2M and as fold change relative to vehicle control, mean±SEM, n=6-10, two-way ANOVA, Sidak's multiple comparison's test, *p≤0.05, **p≤0.01, ****p≤0.0001.
