# Supplementary material for: Type I Interferons Drive the Gastrointestinal Inflammatory Response in a Mouse Model of Parkinson’s Disease
Source: Gastro Hep Adv. 2026 Mar 19;5(6):100929. doi: 10.1016/j.gastha.2026.100929 (PMC13098579; doi:10.1016/j.gastha.2026.100929)
Supplement: Extended PDF [file mmc2.pdf]

Gastro Hep Advances 2026;5:100929

ORIGINAL RESEARCH—BASIC

Type I Interferons Drive the Gastrointestinal Inflammatory Response in a Mouse Model of Parkinson's Disease

Harrison Waters,[1] Shuyan Chen,[1] Elizabeth Vincan,[2,3,4] Dustin J. Flanagan,[5] Renate H. M. Schwab,[6] Peter J. Crack,[1] and Juliet M. Taylor[1]

[1]*Department of Biochemistry and Pharmacology, University of Melbourne, Melbourne, Australia;* [2]*Department of Infectious Diseases, Peter Doherty Institute for Infection and Immunity, University of Melbourne, Melbourne, Australia;* [3]*Victorian Infectious Disease Reference Laboratory, Peter Doherty Institute for Infection and Immunity, Melbourne, Australia;* [4]*Curtin Medical School, Curtin University, Perth, Australia;* [5]*Department of Biochemistry and Molecular Biology, Monash University, Melbourne, Australia; and* [6]*Gulbali Institute, Charles Sturt University, Wagga Wagga, Australia*

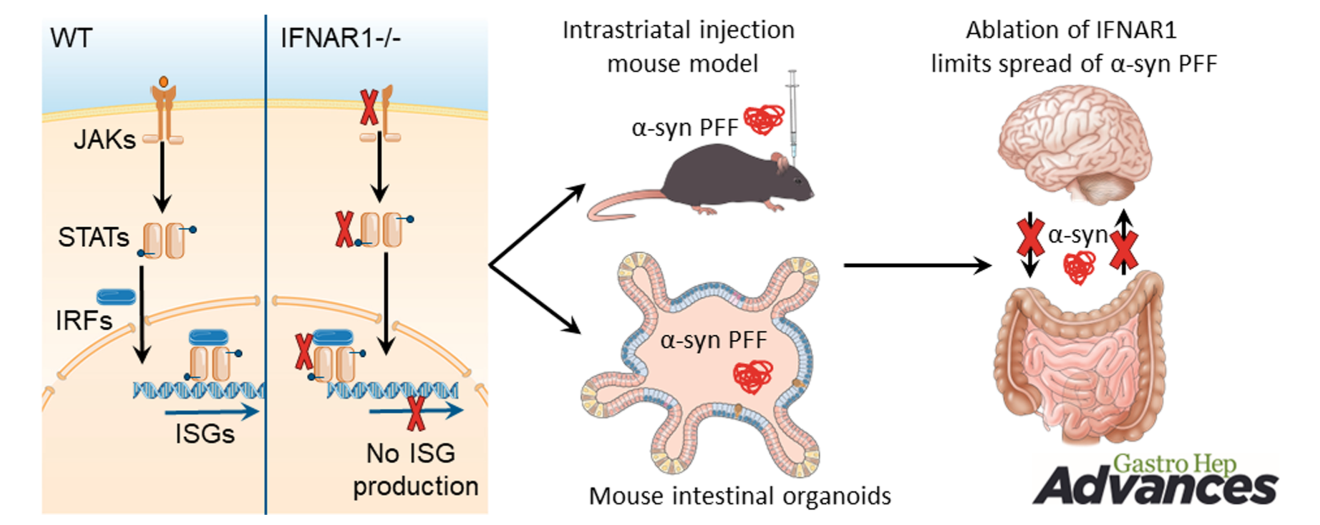

BACKGROUND AND AIMS: Parkinson's disease (PD) is an age-related neurodegenerative disorder characterized by classical motor symptoms due to a loss of dopaminergic neurons in the substantia nigra *pars compacta*. The type I interferons (IFNs) are elevated in the aging brain, and we have implicated them in the neuroinflammatory response in PD. With increasing evidence of gastrointestinal dysfunction in PD patients, this study explored the contribution of the type I IFNs to the transmission of pathology from the brain to the gut in PD. **METHODS:** Young (10–12 weeks) and aged (40–50 weeks) wild-type and IFN alpha receptor (IFNAR)1$^{-/-}$ mice received an intrastriatal injection of human alpha-synuclein (α-syn) preformed fibrils (PFFs) (8 ug) with gut tissue analyzed 6 months postinjection. A mouse intestinal organoid culture model was established to further characterize the α-syn-induced inflammatory response in the gut. **RESULTS:** An intrastriatal injection of human α-syn PFFs was shown to initiate a type I IFN-dependent neuroinflammatory response in the gastrointestinal tract of wild-type mice at 6 months post-injection. This response was attributed to an elevation in type I IFN signaling in aged mice that was absent in the IFNAR1$^{-/-}$ mice. Mouse intestinal organoid cultures confirmed α-syn was taken up by the enteroendocrine cells to induce a type I IFN-mediated pro-inflammatory response that was attenuated in IFNAR1$^{-/-}$ cultures. **CONCLUSION:** This study has confirmed the type I IFNs modulate the α-syn PFF-induced inflammatory response within the gut, potentiating pathology progression along the gut-brain axis. Early intervention of this type I IFN response may be a potential therapeutic target to limit the progression of PD.

Keywords: Parkinson's Disease; Type I Interferons; Alpha-Synuclein; Gut-Brain Axis

Abbreviations used in this paper: **α-syn, alpha-synuclein; BME, biomedical engineered; AP, anterior posterior; CNS, central nervous system; EEC, enteroendocrine cell; ENS, enteric nervous system; GI, gastrointestinal; GIT, gastrointestinal tract; IECs, intestinal epithelial cells; IFN, interferon; IFNAR, interferon alpha receptor; IL, interleukin; i.p., intraperitoneal; IRF, interferon regulatory factor; mIOs, mouse intestinal organoids; PBS, phosphate buffered saline; PBSDT, phosphate buffered saline detergent triton; p-α-syn, phosphorylated α-syn; PD, Parkinson's disease; PFF, preformed fibrils; qPCR, quantitative polymerase chain reaction; SNpc, substantia nigra pars compacta; TGF, transforming growth factor; TNF, tumor necrosis factor; WT, wild type.**

Most current article

Crown Copyright © 2026 Published by Elsevier Inc. on behalf of American Gastroenterological Association Institute. This is an open access article under the CC BY license (http://creativecommons.org/licenses/by/4.0/).

2772-5723
https://doi.org/10.1016/j.gastha.2026.100929

Introduction

Parkinson's disease (PD) is a chronic, progressive neurodegenerative disorder that affects approximately 6.9 million individuals worldwide.[1] Aging is considered the largest risk factor for developing PD, and with an aging population, this number is expected to reach 14.2 million by 2040.[1] The major clinical symptoms of PD are associated with motor dysfunction and include tremor, bradykinesia, postural instability, and muscle rigidity.[2] However, PD patients also present with non-motor symptoms including sensory dysfunction, constipation, speech issues, hypotension, sleep disorders, difficulty in swallowing, cognitive changes, and mental health issues.[3] Some of these symptoms, such as olfactory dysfunction, constipation, and sleep disorders, may present years before diagnosis. Specifically, gastrointestinal (GI) disturbances, such as constipation, can predate common motor symptoms by up to 20 years.[4] The motor symptoms have been attributed to the degeneration of the dopaminergic neurons within the substantia nigra pars compacta (SNpc); however, the precise mechanisms underlying the disease pathogenesis remain unclear.[5] A key pathological hallmark feature within the PD brain is the presence of Lewy bodies containing aggregated alpha-synuclein (α-syn). However, the brain is not the only region of the body that displays α-syn inclusions and Lewy pathology. The identification of α-syn aggregates within the GI tract (GIT)[6] provides a direct link between the gut and brain in the progression of the disease.[7] Specifically, colorectal biopsies of PD patients display phosphorylated α-syn (p-α-syn) pathology, and colonic submucosa p-α-syn levels have been detected in early-stage untreated PD patients.[8,9] Moreover, the expression of p-α-syn in enteric neurons has been shown to correlate with acute and chronic inflammation of the intestinal wall. The density of neutrophils and mononuclear cells is elevated in patient biopsy specimens, with elevated numbers of CD68-positive cells within the lamina propria.[10] This suggests that similar to the central nervous system (CNS), α-syn induces an inflammatory response in the enteric nervous system (ENS); however, the mechanisms involved are not yet fully understood.

Type I interferons (IFNs), a family of pleiotropic cytokines, have been shown to be upregulated in the SNpc of postmortem PD.[11] Furthermore, the presence of this upregulated type I IFN signature in neurodegenerative diseases has been linked to a "reactive" microglial profile. Specifically, single-cell RNA sequencing has identified subpopulations of microglial cells coined "IFN response microglia" that are increased in Alzheimer's disease and in the aged brain.[12,13] The type I IFNs have also been shown to play a role in the neuroinflammatory response in genetic mouse models of PD. PAR$^{-/-}$ and *Pink1*$^{-/-}$ mice display a significant inflammatory phenotype after exhaustive exercise that has been shown to be completely rescued through the loss of STING (Stimulator of Interferon Genes), a downstream mediator of the type I IFN pathway.[14] Furthermore, the loss of dopaminergic neurons in the SNpc and subsequent motor deficits seen in *Prkn*$^{-/-}$ mice were shown to be rescued by targeting STING. In the GIT, the type I IFNs have been shown to regulate intestinal barrier function with the genetic ablation of IFN alpha receptor (IFNAR)1 leading to intestinal hyperplasia, and a lethal loss of intestinal barrier function.[15] With the type I IFNs driving neuroinflammation in the PD brain and also playing a crucial role in maintaining intestinal barrier function, we hypothesized that they modulate the inflammatory response in the gut in PD, thereby contributing to the disease progression along the gut-brain axis. This study confirmed that following an intrastriatal injection of α-syn preformed fibrils (PFFs), an inflammatory response and gut dysfunction were induced that were mediated by the type I IFNs. The modulation of this α-syn-induced inflammatory response was confirmed in vitro using mouse intestinal organoids (mIOs) identifying a potential new pathway to target early non-motor symptoms and disease progression in PD.

Materials and Methods

Animals and Ethics

All experimental procedures were conducted in compliance with the guidelines of the National Health and Medical Research Council of Australia for animal experimentation. All procedures using mice were approved by the Animal Ethics Committee of the University of Melbourne (Ethics #22740). Age-matched male and female C57BL/6J and IFNAR1$^{-/-}$ mice were used for primary and organoid cultures (Ethics #22740). All mice were housed in the Biomedical Sciences Animal Facility, The University of Melbourne. Mice were maintained at 22 ± 2 °C on a 12:12-hour day/night cycle and fed a standard sterile diet of mouse chow with water available ad libitum in standard specific pathogen-free micro isolator cages. All experimental work involving animals followed the Animal Research: Reporting of *In Vivo* Experiments guidelines for animal research.

Fibrilization of α-Synuclein Peptide and Thioflavin-T Assay

One mg α-syn (rPeptide Inc) was diluted to 4 mg/mL in sterile endotoxin-free Dulbecco's phosphate buffered saline (DPBS, Ca^{2+}/Mg^{2+} free) and incubated at 37 °C with agitation on an orbital mixer (1000 rpm) for 7 days. A Thioflavin T assay was used to determine α-syn aggregation using a FLEX3B station (excitation 450 nM, emission 485 nm).

Intrastriatal Injection of Human α-Synuclein Preformed Fibrils

Wild-type (WT, C57Bl/6J) and IFNAR1$^{-/-}$ (young: 8–12 weeks old and aged: 40–50 weeks old, mixed male and female) mice were anesthetized via isoflurane (ISOFLURIN) (2%–5%) inhalation. Mice were transferred onto a digital stereotaxic frame and placed in a nose cone for steady isoflurane inhalation. After drilling a 1 mm burr-hole in the skill, a 5 μL Hamilton syringe with either dPBS (Mg^{2+}/Ca^{2+} free) (GibcoTM, 14200075) or 4 mg/mL α-syn PFF (rPeptide) was used to

deliver 2 μL, at a rate of 0.2 μL/min, into the right dorsal striatum at the following stereotaxic coordinates relative to bregma (mm): −2.0 ML, +0.5 anterior posterior, −3.0 dorso ventral (Supplementary Figure 1). Mice were given a subcutaneous injection of 0.1 mg/kg buprenorphine (120 μL) (Lyppard Australia Pty Ltd) and sterile lactated Ringer's solution (1 mL) (Thermo Fisher Scientific) to facilitate recovery. Wounds were closed using 9 mm Reflex clips. Postoperative mice were closely monitored with a further 0.1 mg/kg buprenorphine administered after 24 hours.

Isolation of Intestinal Crypts

Intestinal crypts were isolated from 8-week-old C57BL/6J and IFNAR1$^{-/-}$ mice using the method of Flanagan et al.[16]

Immunohistochemical Analysis of Mouse Intestinal Organoid Cultures

WT and IFNAR1$^{-/-}$ mIOs were grown in 24-well plates in 50 μL biomedical engineered (BME)-2 spheres. Organoids were fixed with ice-cold 4% w/v paraformaldehyde and gently pipetted up and down to dissociate the BME-2. The organoids were transferred to a 1.5 mL Eppendorf tube and filled to 1 mL with 4% w/v paraformaldehyde, which was then incubated on ice for 30 minutes. The organoids were allowed to pellet via gravity and then washed with 1X PBS (3 × 10 minutes). The PBS was removed and replaced with phosphate buffered saline detergent triton (PBSDT) blocking solution (1X PBS supplemented with 0.1%–1% Triton X-100, 1% dimethyl sulphoxide, 1% BSA, and 1% Goat serum) (500 μL, gentle agitation, room temperature [RT], 2 hours) with the tube allowed to stand for 10 minutes for the organoids to again pellet via gravity. The PBSDT was then removed and replaced with 10% v/v CAS-BlockTM (008120, Invitrogen, Scoresby, VIC, Australia). The blocking agent was then removed and replaced with primary antibodies (Supplementary Table 1) diluted in PBSDT for 24 hours with gentle agitation.

Following an overnight incubation, the cells were washed with PBS/0.1% BSA (4 × 10 minutes), the supernatant was then removed, and the organoids were incubated with secondary antibody (Supplementary Table 1) diluted in PBS/0.1% BSA (gentle agitation, RT, 2 hours). The cells were washed with PBS/0.1% BSA (4 × 10 minutes) before and additional wash with 1 mL 1X PBS for 10 minutes at RT with gentle agitation. To mount slides, the PBS was removed and Vectashield DAPI-containing mounting media (Vector Laboratories) added to pellet. Organoids were mounted using a plastic Pasteur pipette to drop onto a slide before a coverslip was applied. Imaging was conducted on a ZEISS Axio Observer 7.1 microscope and ZEISS LSM880 Airyscan Fast upright confocal microscope.

B16 HEK-Blue Murine Interferon α/β Reporter Cell Line

A B16 HEK-Blue murine IFN α/β reporter cell line (Invivogen) was used as previously described[17] to determine IFN production in mIO cultures.

Cardiac Puncture, Plasma Extraction, and Tissue Collection

WT and IFNAR1$^{-/-}$ mice were anesthetized via an intraperitoneal (i.p.) injection of ketamine (100 mg/kg) (ilium Ketamil) + Xylazine (10 mg/kg) (ilium Xylazil-20). Blood was removed via cardiac puncture and plasma extracted. The colon was removed, then the intestine was excised and separated into duodenum, jejunum, and ileum, with all tissues snap frozen in liquid nitrogen and stored at −80 °C.

RNA Isolation and cDNA Synthesis

RNA was isolated from intestinal tissues (approximately 50–100 mg) using TRIzol (Invitrogen, 15596018). Organoid samples were harvested using Corning Cell Recovery Solution (Corning, 354253) and pelleted before using the RNAeasy mini kit (Qiagen, 74104) as per the manufacturer's instructions. RNA samples were DNase treated with an Ambion TURBO DNA-freeTM Kit (Life Technologies), as per manufacturer's guidelines. RNA was reverse transcribed into cDNA using a high-capacity RNA-to-cDNA Reverse Transcription Kit (4368814, Applied Biosystems).

Real-Time Quantitative Polymerase Chain Reaction

All quantitative polymerase chain reaction (qPCR) was performed in triplicate in standard 384-well plates (4309849, Applied Biosciences, Scoresby, VIC, Australia), with real-time quantitative gene expression determined using TaqMan probes (Supplementary Table 2) and analyzed by the Quant Studio 6 Flex Real-Time PCR System (Invitrogen).

Western Blot Analysis

Western blot analysis was performed on 50 μg of protein. Membranes were incubated with the primary antibodies (Supplementary Table 3) for 24 hours at 4 °C and secondary antibodies for 1.5 hours at RT. Signals detected using an ECL Prime Detection kit (GE Healthcare Life Sciences) and visualized with a ChemiDocTM imaging XRS+ system (Bio-Rad).

Enzyme-Linked Immunosorbent Assay

A mouse tumor necrosis factor (TNF)-alpha Duoset Enzyme-Linked Immunosorbent Assay (R&D Systems, DY410) was used to analyze TNFα levels in mouse plasma and in protein samples extracted from WT and IFNAR1$^{-/-}$ mice gut tissues.

Statistical Analysis

All data presented are expressed as the mean ± SEM. All graphical representations and statistical analysis were carried out using GraphPad PRISM (version 5.03). qPCR data present were analyzed using the Comparative CT Method (ΔΔCt)[18] and is expressed as fold change (compared to vehicle control). B16-Blue assay was analyzed using a 2-way ANOVA, with a Bonferroni post hoc test. Immunohistochemistry comparisons were analyzed via a 2-way Student's *t*-test. For all tests, $P \leq .05$ was accepted as being statistically significant.

Results

Expression of p-α-syn is Elevated in the GIT of Aged WT but not IFNAR1$^{-/-}$ Mice at 6 Months Postinjection (p.i.) of α-syn PFFs Into the Striatum

To determine if age-related increases in type I IFNs influence the brain to gut transmission of α-syn pathology,

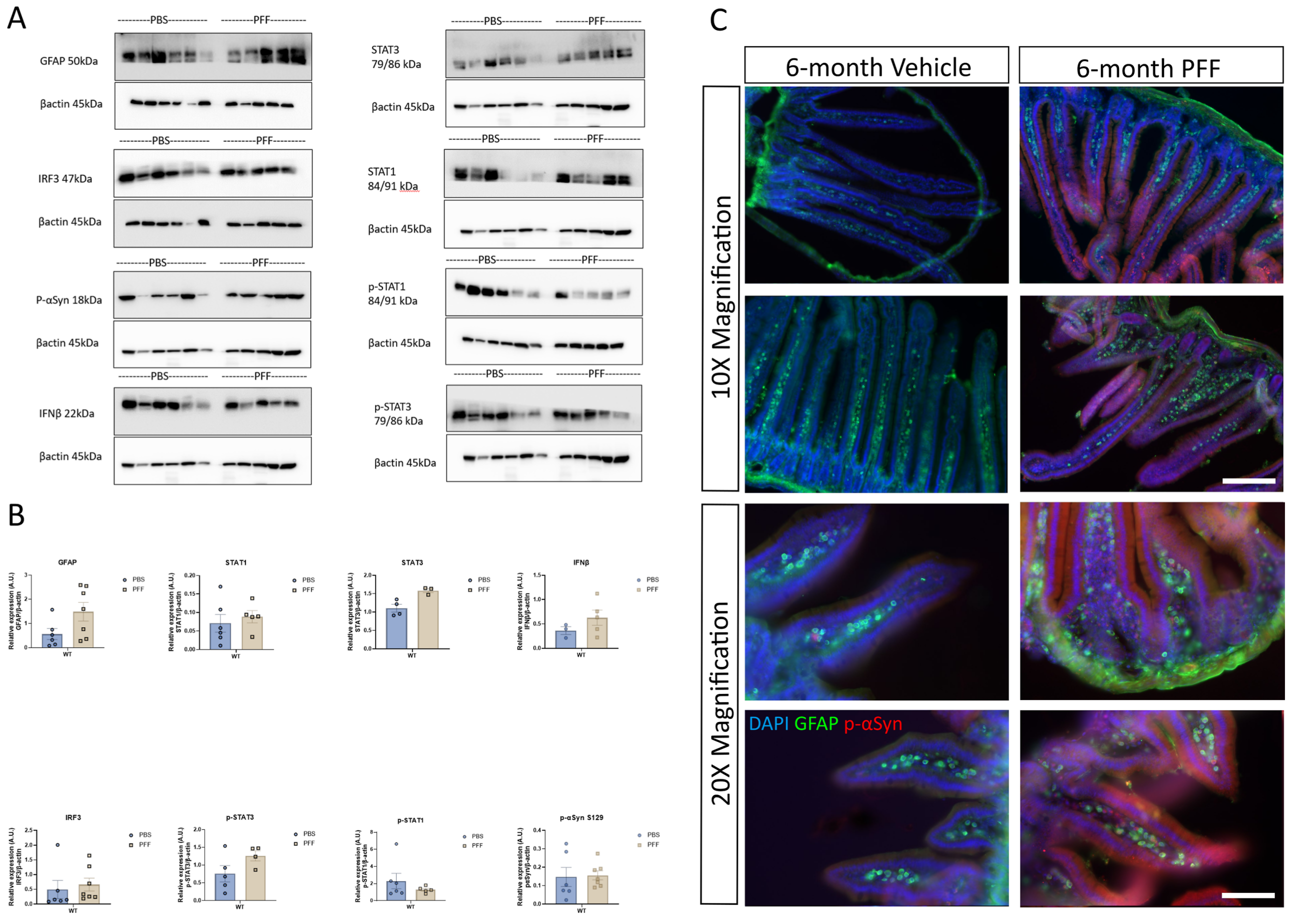

Figure 1. Intrastriatal injection of α-syn PFFs does not induce p-α-syn staining or a proinflammatory response in the GIT of young WT mice at 6 months post injection. Young wild-type mice (10-12 weeks of age at injection) received an intrastriatal injection of α-syn PFFs (or vehicle) before duodenal tissue was excised at 6 months post injection for analysis by western blot (A, B) or immunohistochemistry (C). A, B, Values relative to β-actin were expressed as mean ± SEM; n = 5-6, representative immunoblots of 3 experiments. C, Immunofluorescence images of duodenum from young mice that had received an intrastriatal injection of PBS (vehicle)/a-Syn PFF (PFF) stained for GFAP (green), p-α-syn S129 (red), and DAPI (blue). Scale bar depicts 200 and 100 μm, respectively.

young and aged WT mice received an intrastriatal injection of α-syn PFFs or vehicle (Supplementary Figure 1) and their GI tissues were analyzed 6 months p.i. Young mice that received an intrastriatal injection of α-syn PFFs displayed a similar type I IFN response in the duodenum to that of vehicle controls (Figure 1) with no significant differences in the expression of STAT3, interferon regulatory factor (IRF)3, STAT1, p-Stat1, p-Stat3, or IFN-β. Duodenal expression of GFAP (enteric glial cell marker) and p-α-syn was also unchanged. However, in aged WT mice, IRF3 expression was significantly elevated (0.6-fold, $^{**}P < .01$) in the duodenum of α-syn PFF injected mice, as was IFNβ expression (2-fold, $^{*}P = .05$), when compared to vehicle (Figure 2). There was also an upregulation (2-fold, $^{*}P < .05$) in GFAP expression in α-syn PFF-injected mice compared to vehicle control. Western blot analysis of phosphorylated S129 α-syn levels (detecting both human [exogenous] and mouse [endogenous] α-syn) confirmed a significant upregulation (2-fold, $^{**}P < .01$) in the duodenum of α-syn PFF-injected mice. This upregulation was confirmed by immunofluorescence (Figure 2C) with p-α-syn puncta present in the villus of the duodenum. Interestingly, this α-syn pathology was also seen in young α-syn PFF-injected mice (Figure 1C) that also displayed altered architecture of the intestine. In the young α-syn PFF-injected mice, there was a significant decrease in the villus length accompanied by a significant decrease in crypt length compared to control animals (Supplementary Figure 2). Young PBS-injected mice had a mean villus length of 571 μm compared to young α-syn PFF-injected mice (389.7 μm, $^{****}P < .0001$). An analysis of crypt length also confirmed a reduction in the average crypt length (60.47 μm) in α-syn young PFF-injected mice compared to control mice (63.37 μm). An analysis of the aged cohort identified no significant differences between PBS and α-syn PFF-injected mice (Supplementary Figure 2).

In contrast, there were no significant changes in protein expression of GFAP, IRF3, p-α-syn, and total α-syn in the duodenum of either the young or old IFNAR1$^{-/-}$ mice that had received an intrastriatal injection of α-syn PFFs (compared to vehicle controls).

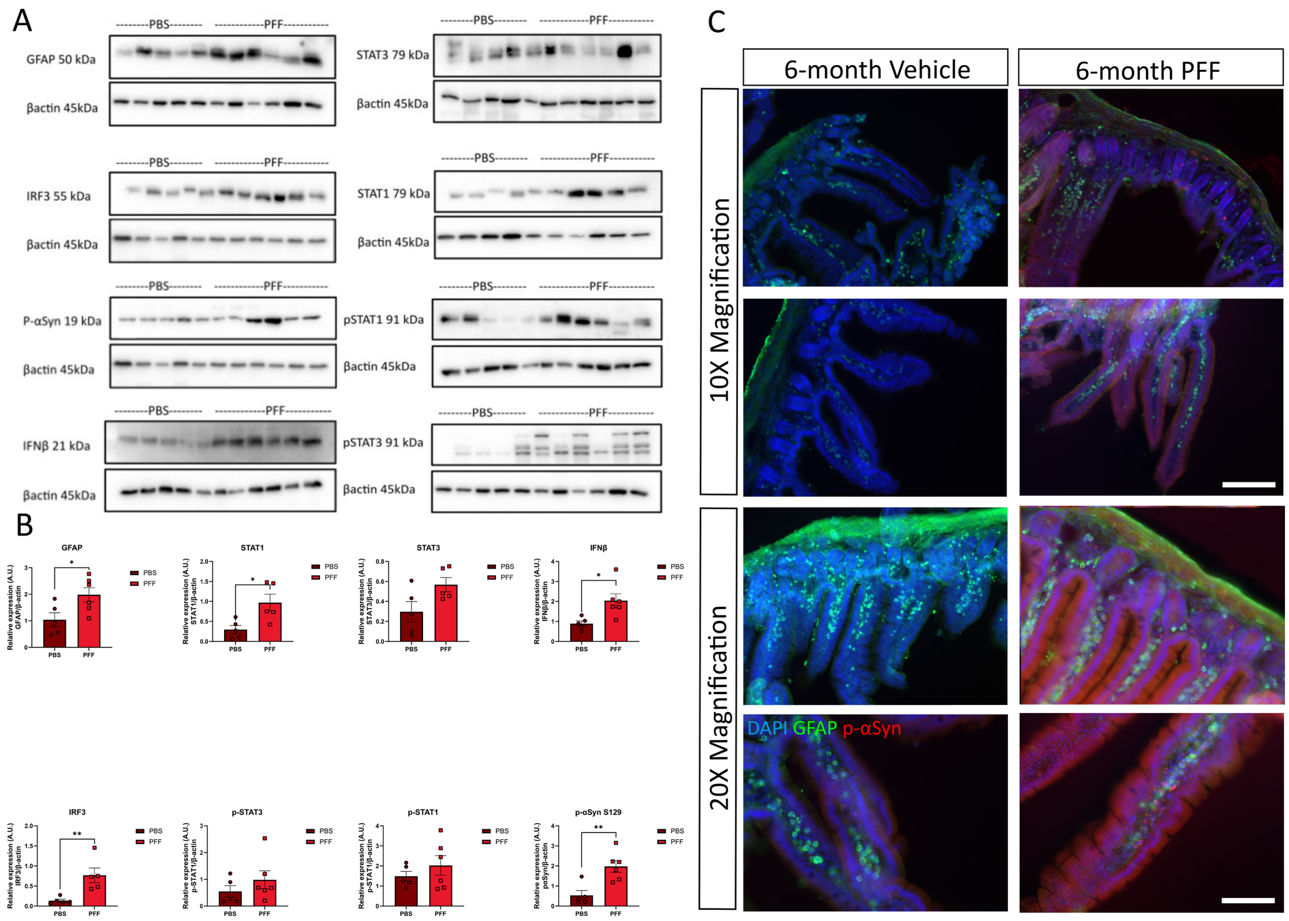

Figure 2. Aging induces p-α-syn staining and a proinflammatory response in the GIT of WT mice at 6 months post intrastriatal injection of α-syn PFFs. Duodenum tissue was excised from aged wild-type mice (40-50 weeks of age at injection) 6 months after receiving an intrastriatal injection of α-syn PFFs or vehicle. A, B, Values expressed relative to b-actin and as mean ± SEM; n = 5-6, images representative of 3 immunoblots. Unpaired Student's *t* test, $^*P \leq .05$, $^{**}P \leq .01$. C, Immunofluorescence images of 30 mM duodenal cryosections from young mice that had received an intrastriatal injection of PBS (vehicle) or a-syn PFFs (PFF) stained for GFAP (green), p-α-syn S129 (red), and DAPI (blue). Scale bar depicts 200 and 100 μm, respectively.

Furthermore, through immunofluorescence analysis, we observed no discernible changes in GFAP expression and limited p-α-syn staining in duodenal sections taken from young and aged IFNAR1$^{-/-}$ mice. There were also no significant differences in the villus and crypt length between vehicle and α-syn PFF-injected mice in either the young or aged cohort (Supplementary Figure 2D). These findings suggest that ablation of IFNAR1 influences the transmission of α-syn from the brain to the gut.

Intrastriatal Injection of α-Synuclein Preformed Fibrils Induces a Type I Interferon-Mediated Pro-inflammatory Response in the Gastrointestinal Tract of Aged Mice

Previous studies have confirmed that following an intrastriatal injection of α-syn PFFs, a pro-inflammatory response is induced in the CNS of mice; however, less is known about the gut response in this model. This study saw no significant differences in mRNA expression of IRF3, IRF7, TNFα, interleukin (IL)-113, transforming growth factor (TGF)-13, and STING between vehicle and α-syn PFF-injected young WT mice (Figure 3). In the colon, α-syn PFFs induced IRF7 expression (compared to vehicle controls); however, the expression of all other genes was unchanged. Conversely, a pro-inflammatory response was detected in duodenum tissue from aged mice at 6 months p.i of α-syn PFFs, with significant upregulations in IRF3 (3-fold, $^*P < .05$), IRF7 (1.8-fold, $^*P < .05$), TNFα (4.5-fold, $^{***}P < .001$), and STING (3.5-fold, $P = <.0001$) (Figure 4). In the jejunum, significant upregulations in IRF3 (3.2-fold, $^{****}P < .0001$), IRF7 (3.3-fold, $^{***}P < .001$), and STING (4.6-fold, $^{**}P < .01$) were detected in the WT α-syn PFF-injected group compared to vehicle controls. mRNA expression in the ileum tissue was unchanged, while the colon displayed significant upregulations in both IL-113 (2.2-fold, $^*P < .05$) and STING (11.4-fold, $^{**}P < .01$). In contrast, at 6 months p.i, the expression of IRF3, IRF7, TNFα, IL-113, TGF13, and STING were unchanged in all regions between α-syn PFF-injected mice and vehicle controls in either the young (Figure 3) or aged (Figure 4) IFNAR1$^{-/-}$ mice.

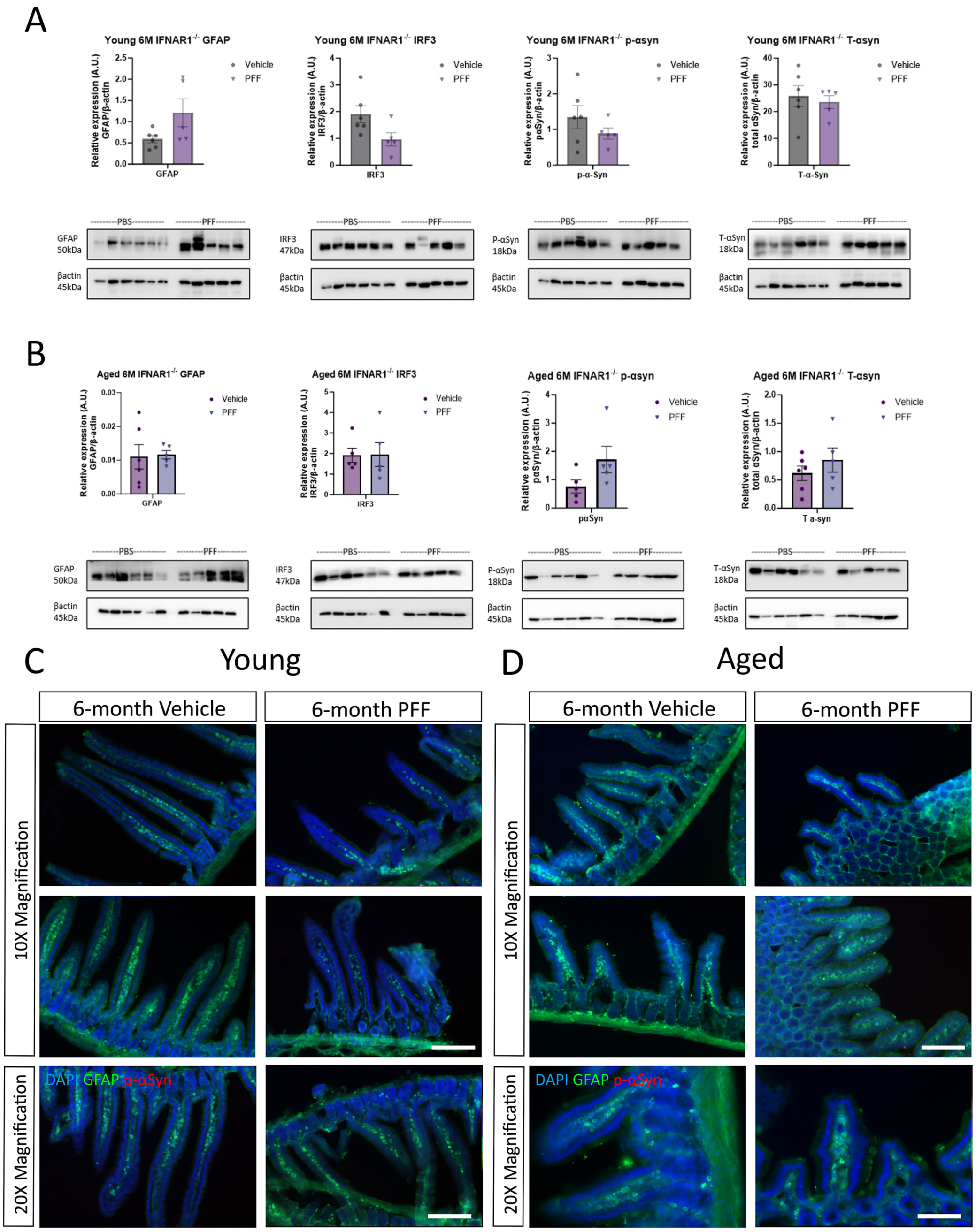

Figure 3. p-α-syn staining and proinflammatory response are absent in the GIT of young and aged IFNAR1$^{-/-}$ mice at 6 months following an intrastriatal injection of α-syn post PFFs. Duodenal tissue was excised from young (10-12 weeks of age at injection) (A, C) and aged (40-50 weeks of age at injection) (B, D) IFNAR1$^{-/-}$ mice that had received an intrastriatal injection of α-syn PFFs or vehicle 6 months previously. A, B, Western blot analysis values expressed relative to β-actin and as mean $\pm$ SEM; n = 5-6, images representative of 3 immunoblots. Immunofluorescence images of 30 μm duodenal cryosections from young mice (C) and aged mice (D), stained for GFAP (green), p-α-syn S129 (red), and DAPI (blue). Scale bar 100 and 200 μm.

α-Synuclein Preformed Fibrils Are Closely Localized With Enteroendocrine Cells Within Mouse Intestinal Organoids

The accumulation of α-syn in neurons and enteric glial cells of the ENS has been reported in GI biopsies from patients with PD with evidence of inflammation also present.9,[19] In both mouse and human in vitro and in vivo models, α-syn has been shown to colocalize with enteroendocrine cells (EECs).[8] To further investigate the α-syn-induced pro-inflammatory response and the contribution of the type I IFNs, an in vitro mIO model was established. Organoids are a medium throughput screening tool to understand the GI epithelium in various disease-specific contexts. In this study, mIOs were used to understand type I IFN's role in modulating the GI epithelial response to α-syn PFFs.

mIOs were treated with 1 μg/mL of human α-syn PFFs for 48 hours, with immunofluorescence confirming WT and

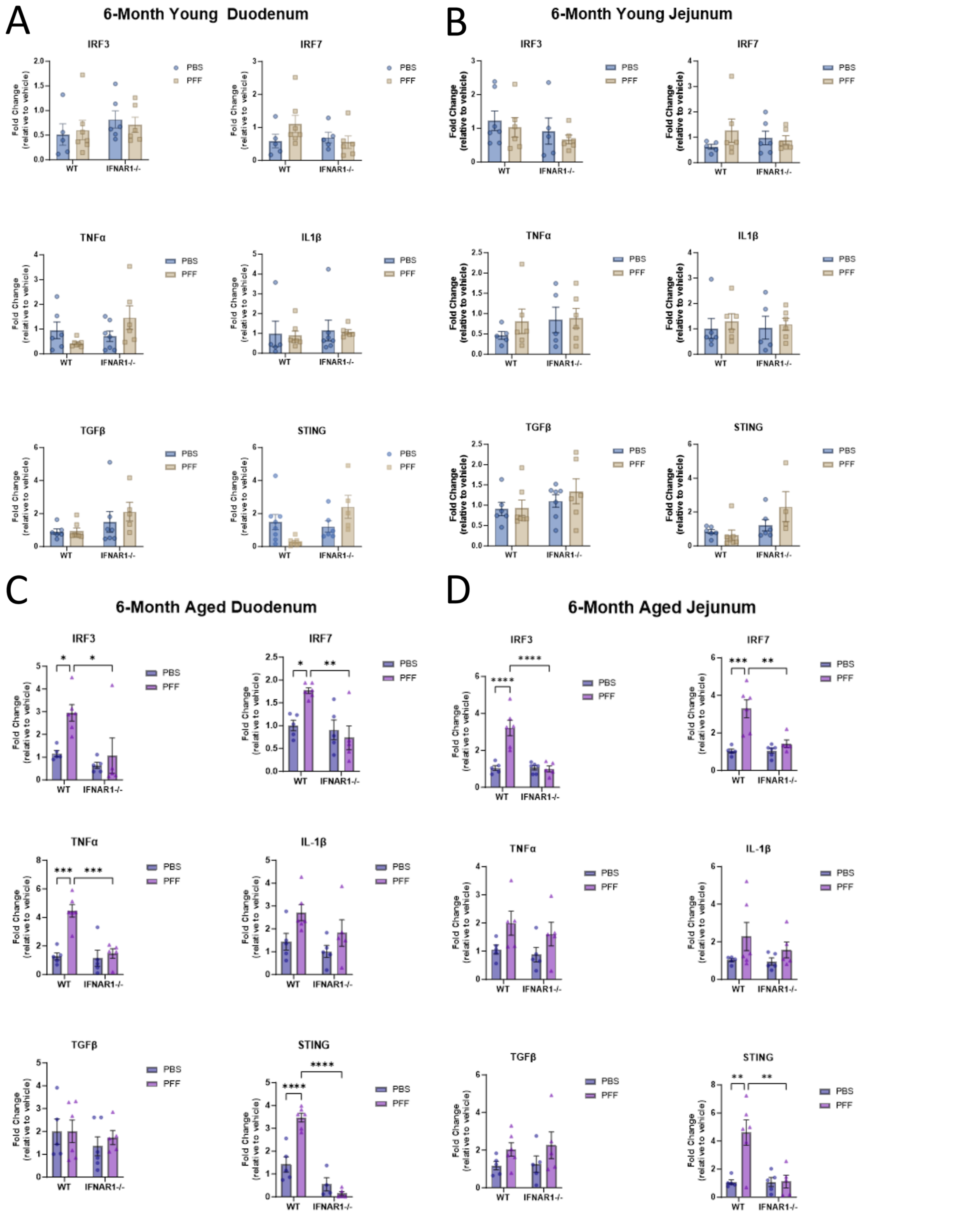

Figure 4. Intrastriatal injection of α-syn PFFs elicits a type I IFN-mediated proinflammatory response in the duodenum and jejunum of aged wild-type but not IFNAR1$^{-/-}$ mice. mRNA expression analysis of duodenum and jejunum tissue from young (10-12 weeks of age at injection) (A, B) and aged (40-50 weeks of age at injection) (C, D) WT and IFNAR1$^{-/-}$ mice that had received an intrastriatal injection of α-syn PFFs or vehicle 6 months previously. Expression levels of IRF-3 & IRF-7, TNFα, IL-1β, TGFβ, and STING were determined as a ratio of beta-2-microglobulin (B2M) expression. Values expressed as fold change (relative to vehicle) and as mean $\pm$ SEM, n = 6-9, 2-way ANOVA, Tukey's multiple comparison test, *$P \leq .05$, **$P \leq .005$, ***$P \leq .001$, ****$P \leq .0001$.

IFNAR1$^{-/-}$ organoids had taken up the exogenous α-syn with total α-syn staining (Figure 5A). Staining with a p-α-syn S129 (human and mouse specific) antibody (Figure 5B and C) was evident within the wall of the intestinal crypt, localizing close to the chromogranin-A positive EECs within the intestinal epithelial cell (IEC) wall. There did not appear

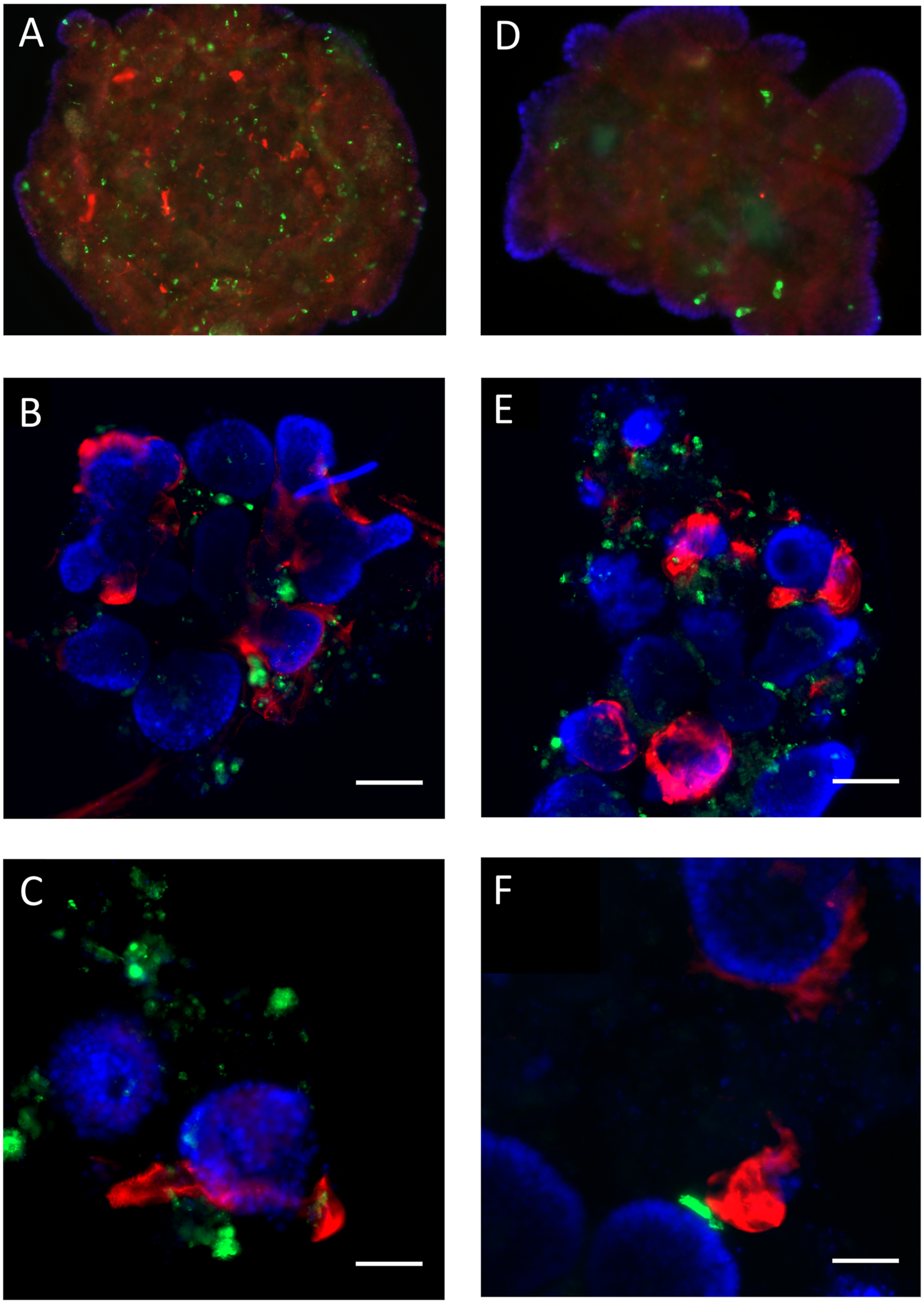

Figure 5. Treatment of wild-type and IFNAR1$^{-/-}$ mIO with α-syn PFFs leads to elevated p-α-syn staining near enteroendocrine cells. Representative immunohistochemical images of wild-type (A, B, and C) and IFNAR1$^{-/-}$ (D, E, and F) mIOs (passage 1, day 7) treated with 1 μg/ml of human α-syn PFFs for 48 hours. A, D, Organoids stained with antibodies to the EEC marker, chromogranin A (green), and total alpha-synuclein (red) and co-tained with DAPI. B, E, Organoids stained with antibodies to the EEC marker, chromogranin A (green), and phospho-Ser129 α-synuclein (red) and costained with DAPI. C and F, Higher power images of (B) and (F) showing localization of α-syn (red) to EEC (green). Scale bar 200 (A, B, D, E) and 100 μm (C, F). Representative images of 3 separate experiments.

to be any gross discernible differences in the localization of α-syn staining between WT and IFNAR1$^{-/-}$ organoids; however, western blot analysis did identify reduced levels in the knockout cultures (Figure 6). Relative to vehicle treated organoids, α-syn PFF-treated WT organoids displayed a significant increase in p-α-syn levels (**$P < .0016$) at 48 hours compared to IFNAR1$^{-/-}$ organoids (Figure 6). This suggests both cultures can take up exogenous α-syn;

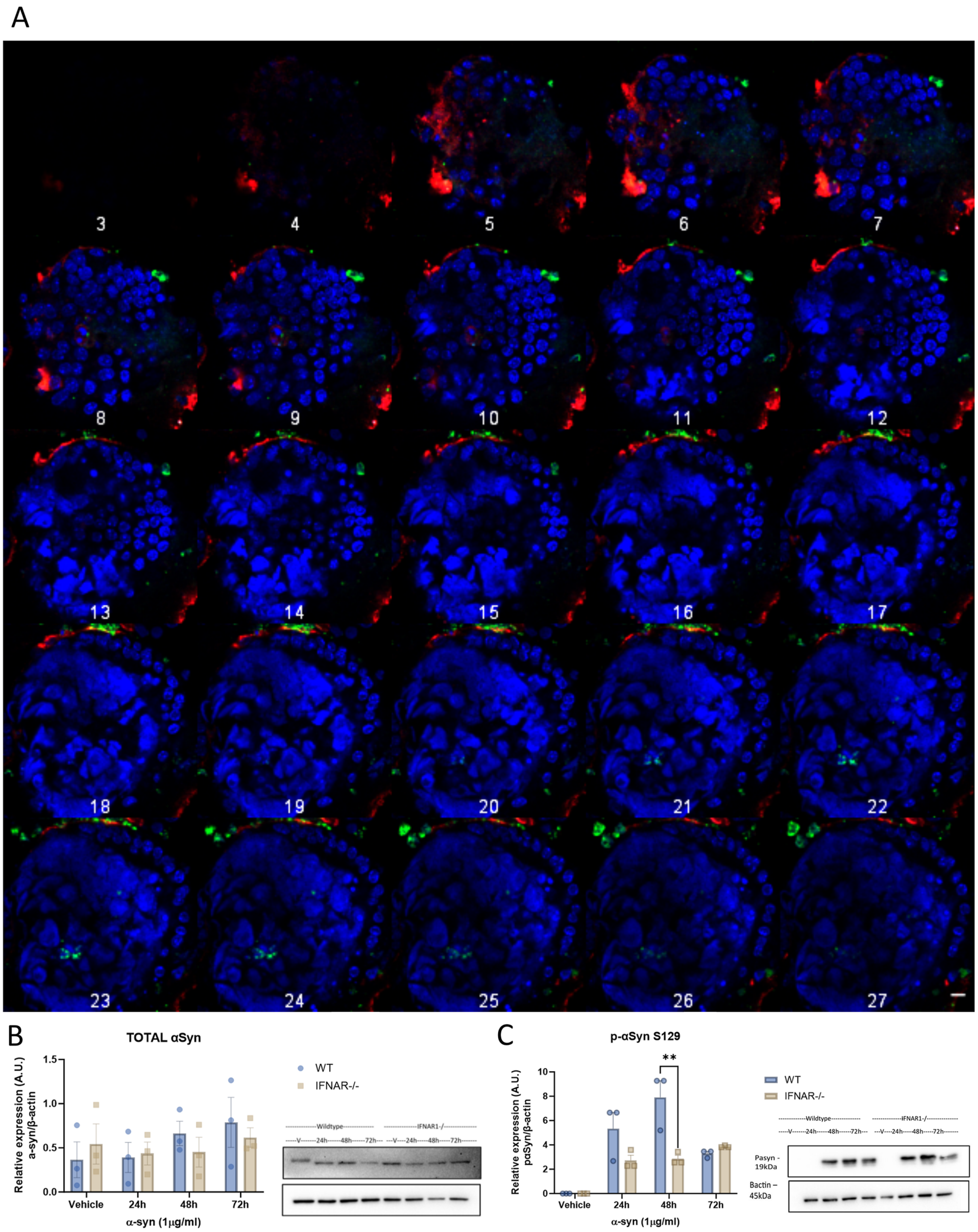

Figure 6. Treatment of wild-type and IFNAR1$^{-/-}$ mIOs with α-syn PFFs leads to an upregulation in p-α-syn levels. A, Wild-type organoids were treated with 1 µg/ml α-syn PFFs for 48 h before being stained with anti-α-synuclein (red), anti-chromogranin A (green), and DAPI (blue). Representative images of a Z-stack showing penetration of α-synuclein into the crypt of the organoid. First slice (1) shows Z position of 0.00 nm; each slice afterward is a Z position of 839.41 nm, with a final Z position of 21.82 µm. B, C, Western blot analysis of WT and IFNAR1$^{-/-}$ organoids treated with 1 µg/ml α-syn PFFs for 24, 48 and 72 h. Blots were stained for total alpha-synuclein (B) or phosphorylated alpha-synuclein (C)) levels with levels expressed as a ratio to β-actin and relative to vehicle control. Two-way ANOVA; data expressed as mean ± SEM, n = 3, $^{**}P \leq .05$.

however, there are differences in its phosphorylation (and potentially aggregation) which may also be true of the endogenous α-syn species.

α-syn PFFs Induce a Pro-inflammatory Response in mIOs That is Type I IFN Dependent

To confirm our in vivo findings and determine if α-syn PFFs could directly induce a type I IFN and pro-inflammatory response in the GIT, WT and IFNAR1$^{-/-}$ mIOs were treated with 1 μg/mL α-syn PFFs for 24–72 hours. qPCR analysis of IFNβ expression levels was increased at 48 hours post α-syn PFFs treatment in WT mIOs (4.8-fold), compared to vehicle controls, with IFNAR1$^{-/-}$ mIOs displaying an attenuated IFNβ response to α-syn PFFs (Figure 7). qPCR analysis also confirmed a significant upregulation in the expression of the

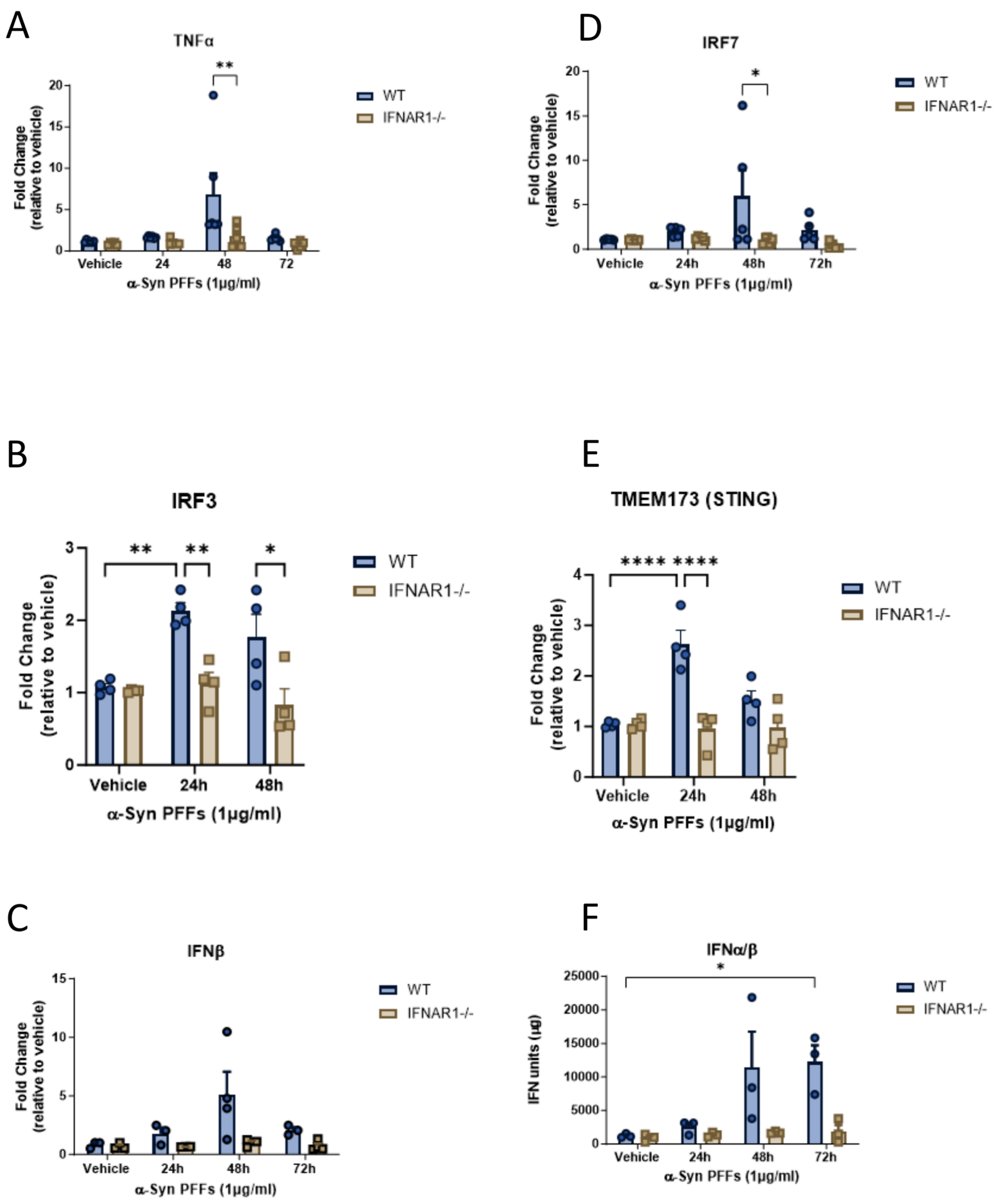

Figure 7. α-syn PFFs induce a type I IFN-mediated proinflammatory response in wild-type mIOs that is attenuated in IFNAR1$^{-/-}$ organoids. Wild-type and IFNAR1$^{-/-}$ mIOs were treated with 1 μg/ml α-syn PFFs for 24, 48, and 72h. A-E, mRNA expression of IRF-3 and IRF-7, TNFα, IFNβ, and STING. Data expressed as fold change relative to B2M and vehicle control. Data expressed as mean ± SEM, two-way ANOVA, Tukey's multiple comparison test, *$P \leq .05$, **$P \leq .01$, ****$P < .0001$. F, A bioactive IFNα/β assay utilising B16 reporter cell lines was used to determine type I FN levels in the media from treated organoids. Data expressed in relative IFN units in μg after adjusting for total protein levels. Data expressed as mean ± SEM, n = 3, two-way ANOVA, Tukey's multiple comparison test, *$P \leq .05$.

downstream mediator, IRF7 in WT mIOs at 48 hours (12.7-fold), which was attenuated in the IFNAR1$^{-/-}$ organoids (0.95-fold) (Figure 7D). IRF3 (2.0-fold, *$P < .05$) and STING (2.5-fold, ****$P < .0001$) mRNA expression was upregulated at 24 hours, with both attenuated in the IFNAR1$^{-/-}$ organoids (Figure 7B and E). An upregulation in the type I IFNs was confirmed using a B16-BlueTM IFN reporter assay. Wildtype organoids displayed robust increases in bioactive IFNα/β expression at 48 hours (11,369.40 ± 5421.14) and 72 hours (12,234.52 ± 2051.19), this response was diminished in the IFNAR1$^{-/-}$ cultures at 48 hours (1707.81 ± 91.69, *$P < .05$) and 72 hours (1848.14 ± 833.73, *$P < .05$) respectively (Figure 7F). This attenuated type I IFN response in the IFNAR1$^{-/-}$ mIOs correlated with a reduced pro-inflammatory response. Wildtype organoids treated with α-syn PFFs displayed an upregulation in TNFα expression at 48 hours (compared to vehicle) (5.200 ± 1.882), with this response attenuated

in the IFNAR1$^{-/-}$ cultures (1.531 ± 0.589, $*P \leq .05$) (Figure 7A).

Discussion

This study provides evidence for the type I IFNs in modulating the transmission of α-syn pathology from the CNS to the GIT in a mouse model of PD. This transmission was age dependent with the type I IFN-mediated inflammatory phenotype only seen in WT mice. Type I IFNs are known to be elevated in aged mice and humans and have previously been linked to aging-related cognitive decline.[13] Accordingly, we and others have identified the type I IFNs in both postmortem human AD and PD brains and in mouse models of the diseases.[11,12,20] Although the function of the type I IFNs in the GIT under both homeostatic conditions[21] and following injury is well established,[22] this study is the first to implicate the type I IFNs in the GIT pathology in PD.

We have previously confirmed an early upregulation in CNS expression of the type I IFNs in the MPTP mouse model of PD.[11] IFNAR1$^{-/-}$ mice display reduced neuroinflammation and neuroprotection against nigral cell death and locomotor deficits induced by MPTP. The MPTP model has several limitations; it is an acute model involving the i.p. injection of MPTP (4×2-hourly doses) and the subsequent analyses at 21 days p.i., therefore it is not a true representation of the human disease progression. The MPTP model also displays an absence of α-syn pathology within the CNS. The i.p injection of MPTP precludes any specific brain-gut transmission to be identified; therefore, for this study, we chose to utilize the intrastriatal α-syn PFF model that induces a significant CNS pro-inflammatory response, p-α-syn pathology, nigrostriatal pathway loss, and behavioral deficits.[23–25] It should be noted that these studies have predominately focused on the CNS pathology and subsequent motor symptoms that are seen in the human disease. We hypothesized that the type I IFN-mediated inflammatory response that we reported in the CNS[11] would also be a driver of the GIT pathology and non-motor symptoms that occur in PD.

There is supporting evidence for a relationship between α-syn, inflammation, and disease progression along the gut-brain axis in several PD mouse models. Kishimoto et al[26] reported chronic intestinal inflammation in α-syn mutant mice that presented with accelerated brain neuropathology and motor dysfunction. Furthermore, increased markers of inflammation (TNFα, IL-6, IL-1β, and IL-10) were seen in both the colon and the brain, consistent with the retrograde transneuronal propagation of α-syn pathology and neuroinflammation from the gut to the brain. These findings were corroborated by Kim et al,[27] who reported the ability of α-syn PFFs to induce inflammation and pathology through the transportation of aggregated α-syn from the gut to the brain. Other studies have confirmed that inoculation of α-syn PFFs into the GIT of both mice and rats leads to aggregation of α-syn primarily near the injection site but also in areas of the brain.[28,29] In addition, Challis et al[30] reported a more severe phenotype in aged mice that received a gut injection of α-syn PFFs. Specifically, they found increased p-α-syn staining in the brainstem and midbrain, compared to young, injected mice. Moreover, the researchers found that aged mice also presented with decreased striatal dopamine when compared to young, injected mice.

To our knowledge, however, this is the first time it has been shown that aged mice exhibit greater brain-gut transmission of α-syn pathology and an exacerbated pro-inflammatory GI phenotype. Immunohistochemical and western blot analyses identified elevated phosphorylated S129 α-syn levels in the duodenum of aged mice (Figure 2) that were not seen in young mice (Figure 1) at 6 months p.i. of α-syn PFFs into the striatum. This p-α-syn antibody detected both human and mouse p-α-syn on S129A. Similarly, an elevated type I IFN and pro-inflammatory response was only seen in the duodenum of aged WT mice. Increases in pro-inflammatory cytokines (IFNγ and TNFα) are upregulated in the human PD GIT and have been reported in the GIT of other models of PD including peripheral MPTP injections; however, this was the first report confirming the type I IFNs involvement in this response. Type I IFNs are elevated in the aging gut, and in an analysis of mIO cultures, it was specifically shown that the IFN-Stat1 axis drives aging-associated loss of intestinal tissue homeostasis and regeneration.[31] Furthermore, the group identified an increase in pro-inflammatory cells in the lamina propria of the aged intestine that perpetuated the induction of Stat1 activity in intestinal stem cells priming the aberrant differentiation and increase in antigen presentation on the IECs.

The phenotype seen in the aging mouse is indicative of that reported in PD patients, with a worsening of GI barrier function with age.[32] We postulated that the elevated type I IFNs in the aging gut were contributing to the exacerbated pro-inflammatory response following an intrastriatal injection of α-syn PFFs. Indeed, IFNAR1$^{-/-}$ mice displayed an attenuated pro-inflammatory and type I IFN response with reduced α-syn staining in the duodenum. Intestinal inflammation has been previously linked to changes in the morphology of villus and crypts within the small intestine[33,34]; however, we only saw these changes in our young mice. In aged mice, no significant differences between treatment groups were identified despite aged mice having lower villus size overall (Supplementary Figure 2B). However, in the young mice, villus and crypt length in the upper duodenum of α-syn PFF-injected WT mice was reduced compared to their vehicle controls. There was no significant difference in the IFNAR1$^{-/-}$ duodenum (Supplementary Figure 2D). This finding may contribute to our understanding of the type I IFN's role in the GI epithelium. Type I IFNs have been linked to intestinal morphology and the composition of the gut microbiota in mice. A study from Brodziak et al[35] has shown that the colonic mucosa expression of IFN-related genes was differentially expressed in separate mouse strains after

colonization of commensal microbiota. Furthermore, an analysis of the microbiota of mice with selective type I IFN deletion in IECs depicted changes in the composition of microbiota, Paneth cell abundance, and ability of the epithelium to regenerate.[36] In addition, Thompson et al[37] reported that mice lacking IRF9, which are unable to respond to type I IFNs, displayed a higher temporal variation of microbiota, accompanied by an increase in the number of T cells and neutrophils in the GIT. In addition to these changes, it has been presented that in the brain throughout aging there is a type I IFN signature that is shown to negatively affect brain function.[13] The study reported that blocking type I IFN signaling within the aged brain partially restored cognitive function and hippocampal neurogenesis. In the ENS, it has been shown that impaired type I IFN induction in aging impairs viral clearance and promotes immunopathology following infection.[38] These findings taken together may elucidate a functional decline of both the brain and the gut in aging.

To further determine the effects exogenous α-syn PFFs have on intestinal morphology, mIOs were utilized. Immunohistochemistry confirmed similar staining patterns of human α-syn PFFs in both WT and IFNAR1$^{-/-}$ mIOs, with close localization of anti-α-syn staining with chromogranin-A staining of EECs.

EECs form the largest endocrine organ in the body and play a key role in the control of GI secretion and motility, regulation of food intake, and glucose homeostasis.[39] They also have an intrinsic connection to the gut microbiota and have been shown to be modulated in response to short-chain fatty acids and lipopolysaccharide.[40] The EECs are complex sensory sentinels of the intestinal environment. They are involved in the detection of inflammation via toll-like receptors[41] and microbiome dysbiosis through microbial metabolites.[42] In addition, EECs increase crypt cell proliferations via increased growth factor release.[43] EECs also influence cytokine and antimicrobial production from enterocytes and Paneth cells, while maintaining tight junctions of the epithelium.[44,45] It has also recently been reported that EECs contain a neuropod that innervates toward the ENS and glia. This neuropod is hypothesized to enable bidirectional communication between EECs and the ENS.[40] Clinical evidence in human patients suffering from IBS has reported that the T-cell-mediated inflammation present in GIT is coupled with a 5-fold increase in EEC cell number and changes in gut permeability.[46] Similarly, increased EECs have also been demonstrated in celiac patients and are associated with inflammation of the intestine.[47] Recently, the contribution of this secretory cell type to the PD gut disturbances has been proposed. Chandra et al[8] identified α-syn staining within EECs which directly connected to α-syn-containing nerves—thus forming a neural circuit between the brain and gut in which toxins and other environmental influences may influence α-syn folding in the EECs. They suggested that this may provide a possible pathway for misfolded α-syn propagation from the gut epithelium to cause inflammation. Our findings showing α-syn PFFs inducing a pro-inflammatory response (increased expression of TNFα) in mIO cultures support this pathogenic mechanism.

The findings from our in vitro cultures corroborate our mouse model studies, identifying the type I IFNs as key mediators of the α-syn induced pro-inflammatory response in the GIT. The pro-inflammatory response induced by α-syn was coupled with an increase in both IFNβ and IRF7 levels indicating activation of the type I IFN signaling pathway. It has been suggested that IRF7 plays a key modulatory role in the homeostatic balance of inflammation within the gut. Recently, Qing and Liu[48] postulated that IRF7 may play a pro-inflammatory role in promoting expression of IL-5 and IL-13 induced by BCL11B or directly promoting the expression of inflammatory cytokines and chemokines such as IL-6, TGF-β, CCL5, and CXCL10. Furthermore, IRF7 KO mice when compared to WT, presented with decreased IL-6 gene expression levels, lower expression of profibrotic factors in fibroblasts, less subcutaneous thickness, and milder inflammation response to bleomycin stimulation.[49]

The type I IFNs orchestrate a series of intracellular events in immune cells and IECs that leads to the resolution of inflammation and allow the regeneration of the intestinal epithelium and restoration of the gut barrier.[21] Type I IFNs are secreted by dendritic cells and phagocytes in response to microbial attack/tissue injury.[50] Furthermore, type I IFNs may act on Paneth cells to restrict proliferation and favor their differentiation to establish gut barrier permeability.[21] However, our findings suggest that in response to α-syn deposition, the type I IFNs are instrumental to both the acute and chronic inflammatory response in the GIT. It has been shown that IECs themselves are capable of producing type I IFNs, as well as other cytokines and chemokines.[51] In the DSS model of colitis, mice with attenuated type I IFN signaling display an expansion of Paneth cell numbers and hyperproliferation of the intestinal epithelium, when compared to WT littermates.[36] We identified significantly decreased intestinal villus length in our α-syn PFF-injected IFNAR1$^{-/-}$ mice; however, this finding was only seen in young, and not aged mice. It is feasible that the changes in morphology in the guts of aged animals are less responsive to α-syn, when compared to young mice. This hypothesis is supported by animal studies using the α-syn PFF model.[30] It has been postulated that younger adult and aged mice have a differing response due to protein homeostatic function declining in the gut with age.[52] In addition, the ability to eliminate α-syn aggregates is expected to be affected to promote gut-brain transmission and sensorimotor deficits.[30]

One of the limitations of this study is that due to the age and accompanying health problems of our aged cohort, the timeline was unable to be extended beyond the 6-month analysis to also confirm α-syn propagation and pathology in our young cohort. In comparison, in assessing gut-brain transmission, Kim et al[27] analyzed brains for α-syn pathology at 10 months p.i. It should be noted that when validating our model, small but significant motor deficits as assessed by

Digigait analysis were identified in the young WT but not IFNAR1$^{-/-}$ mice at 3 months p.i., (Chen et al, unpublished data). This study chose to focus solely on the GIT in this model and confirmed the type I IFNs regulate the α-syn-induced neuroinflammatory response implicated in the propagation of pathology along the gut-brain axis. Age-related elevations in type I IFNs were shown to exacerbate this disease progression, supporting previous studies implicating the type I IFNs in neuropathologies such as AD.[13]

To our knowledge, this study is the first to use mIO cultures as a model of GIT dysfunction in PD and allowed us to directly confirm an α-syn-induced inflammatory response. The use of intestinal organoids in the PD field has not been reported until a recent review paper by Reiner et al[53], which discussed future possibilities of using multi-organ systems to model both peripheral and central aspects of PD. The potential of these systems to facilitate the study of the gut-brain axis in PD in vitro and their use for more high-throughput therapeutic approaches to target aspects of disease was discussed. More recently, a paper by Chandra et al[54] used mIOs from A53T synuclein mice with isolated nodose ganglion neurons from SNCA$^{-/-}$ mice to understand the transfer of α-syn between these cell types. They confirmed human α-syn transferred between the mIOs and vagal neurons in this co-culture model, with α-syn pathology moving through vagal neuron processes from cell-to-cell. Our findings specifically demonstrate that α-syn PFFs are taken up by mIOs, and mIOs mount a type I IFN-mediated immune response to PFFs. Our study is, to our knowledge, the first instance it has been shown that mIOs produce an inflammatory response to α-syn PFF.

Our findings identify a key role for the type I IFNs within the intestinal epithelium in modulating inflammatory mediators in response to toxins or stressors such as α-syn. In conclusion, our data confirms that α-syn pathology in the brain is sufficient to drive an inflammatory response in the GIT, that is exacerbated by age-related elevations in the type I IFNs. Future in vivo studies will build on our findings from our mIO cultures to determine if the α-syn association with the intestinal epithelium and subsequent type I IFN inflammatory response can be therapeutically targeted to limit the disease progression and non-motor symptoms in PD.

Supplementary Materials

Material associated with this article can be found, in the online version, at https://doi.org/10.1016/j.gastha.2026.100929.

References

1. Dorsey ER, Bloem BR. The Parkinson pandemic—a call to action. JAMA Neurol 2018;75:910.
2. Rodriguez-Oroz MC, Jahanshahi M, Krack P, et al. Initial clinical manifestations of Parkinson's disease: features and pathophysiological mechanisms. Lancet Neurol 2009;8:11281139.
3. Hibar DP, Westlye LT, Doan NT, et al. Cortical abnormalities in bipolar disorder: an MRI analysis of 6503 individuals from the ENIGMA Bipolar Disorder Working Group. Mol Psychiatry 2018;23:932–942.
4. Savica R, Carlin JM, Grossardt BR, et al. Medical records documentation of constipation preceding Parkinson disease: a case-control study. Neurology 2009;73:1752–1758.
5. Surmeier DJ. Determinants of dopaminergic neuron loss in Parkinson's disease. FEBS J 2018;285:3657–3668.
6. Cersosimo MG. Gastrointestinal biopsies for the diagnosis of alpha-synuclein pathology in Parkinson's disease. Gastroenterol Res Pract 2015;2015:476041.
7. Liddle RA. Parkinson's disease from the gut. Brain Res 2018;1693:201–206.
8. Chandra R, Hiniker A, Kuo YM, et al. Alpha-synuclein in gut endocrine cells and its implications for Parkinson's disease. JCI Insight 2017;2:e92295.
9. Shannon KM, Keshavarzian A, Mutlu E, et al. Alpha-synuclein in colonic submucosa in early untreated Parkinson's disease. Mov Disord 2012;27:709–715.
10. Stolzenberg E, Berry D, Yang D, et al. A role for neuronal alpha-synuclein in gastrointestinal immunity. J Innate Immun 2017;9:456–463.
11. Main BS, Zhang M, Brody KM, et al. Type-1 interferons contribute to the neuroinflammatory response and disease progression of the MPTP mouse model of Parkinson's disease. Glia 2016;64:1590–1604.
12. Roy ER, Wang B, Wan YW, et al. Type I interferon response drives neuroinflammation and synapse loss in Alzheimer disease. J Clin Invest 2020;130:1912–1930.
13. Baruch K, Deczkowska A, David E, et al. Aging. Aging-induced type I interferon response at the choroid plexus negatively affects brain function. Science 2014;346:89–93.
14. Sliter DA, Martinez J, Hao L, et al. Parkin and PINK1 mitigate STING-induced inflammation. Nature 2018;561:258–262.
15. Katlinskaya YV, Katlinski KV, Lasri A, et al. Type I interferons control proliferation and function of the intestinal epithelium. Mol Cell Biol 2016;36:1124–1135.
16. Flanagan DJ, Schwab RHM, Tran BM, et al. Isolation and culture of adult intestinal, gastric, and liver organoids for cre-recombinase-mediated gene deletion. Methods Mol Biol 2019;1576:123–133.
17. Moore Z, Mobilio F, Walker FR, et al. Abrogation of type-I interferon signalling alters the microglial response to Abeta(1-42). Sci Rep 2020;10:3153.
18. Livak KJ, Schmittgen TD. Analysis of relative gene expression data using real-time quantitative PCR and the 2(-Delta Delta C(T)) Method. Methods 2001;25:402–408.
19. Sanchez-Ferro A, Rabano A, Catalan MJ, et al. In vivo gastric detection of alpha-synuclein inclusions in Parkinson's disease. Mov Disord 2015;30:517–524.
20. Minter MR, Moore Z, Zhang M, et al. Deletion of the type-1 interferon receptor in APPSWE/PS1DeltaE9 mice preserves cognitive function and alters glial phenotype. Acta Neuropathol Commun 2016;4:72.

21. Kotredes KP, Thomas B, Gamero AM. The protective role of type I interferons in the gastrointestinal tract. Front Immunol 2017;8:410.
22. McElrath C, Espinosa V, Lin JD, et al. Critical role of interferons in gastrointestinal injury repair. Nat Commun 2021;12:2624.
23. Luk KC, Kehm V, Carroll J, et al. Pathological alpha-synuclein transmission initiates Parkinson-like neurodegeneration in nontransgenic mice. Science 2012; 338:949–953.
24. Luk KC, Kehm VM, Zhang B, et al. Intracerebral inoculation of pathological alpha-synuclein initiates a rapidly progressive neurodegenerative alpha-synucleinopathy in mice. J Exp Med 2012;209:975–986.
25. Paumier KL, Luk KC, Manfredsson FP, et al. Intrastriatal injection of pre-formed mouse α-synuclein fibrils into rats triggers α-synuclein pathology and bilateral nigrostriatal degeneration. Neurobiol Dis 2015; 82:185–199.
26. Kishimoto Y, Zhu W, Hosoda W, et al. Chronic mild gut inflammation accelerates brain neuropathology and motor dysfunction in α-synuclein mutant mice. Neuromolecular Med 2019;21:239–249.
27. Kim S, Kwon SH, Kam TI, et al. Transneuronal propagation of pathologic α-synuclein from the gut to the brain models Parkinson's disease. Neuron 2019; 103:627–641.e7.
28. Holmqvist S, Chutna O, Bousset L, et al. Direct evidence of Parkinson pathology spread from the gastrointestinal tract to the brain in rats. Acta Neuropathol 2014; 128:805–820.
29. Uemura N, Yagi H, Uemura MT, et al. Inoculation of alpha-synuclein preformed fibrils into the mouse gastrointestinal tract induces Lewy body-like aggregates in the brainstem via the vagus nerve. Mol Neurodegener 2018;13:21.
30. Challis C, Hori A, Sampson TR, et al. Gut-seeded α-synuclein fibrils promote gut dysfunction and brain pathology specifically in aged mice. Nat Neurosci 2020; 23:327–336.
31. Omrani O, Krepelova A, Rasa SMM, et al. IFNgamma-Stat1 axis drives aging-associated loss of intestinal tissue homeostasis and regeneration. Nat Commun 2023;14:6109.
32. van ISCD, Derkinderen P. The intestinal barrier in Parkinson's disease: current state of knowledge. J Parkinsons Dis 2019;9:S323–S329.
33. Parker A, Vaux L, Patterson AM, et al. Elevated apoptosis impairs epithelial cell turnover and shortens villi in TNF-driven intestinal inflammation. Cell Death Dis 2019;10:108.
34. Wilson FD, Cummings TS, Barbosa TM, et al. Comparison of two methods for determination of intestinal villus to crypt ratios and documentation of early age-associated ratio changes in broiler chickens. Poult Sci 2018;97:1757–1761.
35. Brodziak F, Meharg C, Blaut M, et al. Differences in mucosal gene expression in the colon of two inbred mouse strains after colonization with commensal gut bacteria. PLoS One 2013;8:e72317.
36. Tschurtschenthaler M, Wang J, Fricke C, et al. Type I interferon signalling in the intestinal epithelium affects Paneth cells, microbial ecology and epithelial regeneration. Gut 2014;63:1921–1931.
37. Thompson CL, Hofer MJ, Campbell IL, et al. Community dynamics in the mouse gut microbiota: a possible role for IRF9-regulated genes in community homeostasis. PLoS One 2010;5:e10335.
38. Feng E, Balint E, Poznanski SM, et al. Aging and interferons: impacts on inflammation and viral disease outcomes. Cells 2021;10:708.
39. Latorre R, Sternini C, De Giorgio R, et al. Enteroendocrine cells: a review of their role in brain-gut communication. Neurogastroenterol Motil 2016;28:620–630.
40. Gribble FM, Reimann F. Function and mechanisms of enteroendocrine cells and gut hormones in metabolism. Nat Rev Endocrinol 2019;15:226–237.
41. Selleri S, Palazzo M, Deola S, et al. Induction of pro-inflammatory programs in enteroendocrine cells by the Toll-like receptor agonists flagellin and bacterial LPS. Int Immunol 2008;20:961–970.
42. Samuel BS, Shaito A, Motoike T, et al. Effects of the gut microbiota on host adiposity are modulated by the short-chain fatty-acid binding G protein-coupled receptor, Gpr41. Proc Natl Acad Sci U S A 2008; 105:16767–16772.
43. Ørskov C, Hartmann B, Poulsen SS, et al. GLP-2 stimulates colonic growth via KGF, released by subepithelial myofibroblasts with GLP-2 receptors. Regul Pept 2005; 124:105–112.
44. Hougaard DM, Larsson LI. Carboxypeptidase E in rat antropyloric mucosa: distribution in progenitor and mature endocrine cell types. Histochem Cell Biol 2004; 121:55–61.
45. Dong CX, Zhao W, Solomon C, et al. The intestinal epithelial insulin-like growth factor-1 receptor links glucagon-like peptide-2 action to gut barrier function. Endocrinology 2014;155:370–379.
46. Spiller RC, Jenkins D, Thornley JP, et al. Increased rectal mucosal enteroendocrine cells, T lymphocytes, and increased gut permeability following acute Campylobacter enteritis and in post-dysenteric irritable bowel syndrome. Gut 2000;47:804–811.
47. El-Salhy M. Invited review-the nature and implication of intestinal endocrine cell changes in coeliac disease. Histol Histopathol 1998;13:1069–1076.
48. Qing F, Liu Z. Interferon regulatory factor 7 in inflammation, cancer and infection. Front Immunol 2023;14: 1190841.
49. Wu M, Skaug B, Bi X, et al. Interferon regulatory factor 7 (IRF7) represents a link between inflammation and fibrosis in the pathogenesis of systemic sclerosis. Ann Rheum Dis 2019;78:1583–1591.
50. Montoya M, Schiavoni G, Mattei F, et al. Type I interferons produced by dendritic cells promote their phenotypic and functional activation. Blood 2002; 99:3263–3271.
51. Lozhkov AA, Klotchenko SA, Ramsay ES, et al. The key roles of Interferon lambda in human molecular defense against respiratory viral infections. Pathogens 2020; 9:989.
52. Douglas PM, Dillin A. Protein homeostasis and aging in neurodegeneration. J Cell Biol 2010;190:719–729.

53. Reiner O, Sapir T, Parichha A. Using multi-organ culture systems to study Parkinson's disease. Mol Psychiatry 2021;26:725–735.
54. Chandra R, Sokratian A, Chavez KR, et al. Gut mucosal cells transfer alpha-synuclein to the vagus nerve. JCI Insight 2023;8:e172192.

Received October 1, 2025. Accepted March 12, 2026.

Correspondence:
Address correspondence to: Peter J. Crack, BSc (Hons), PhD, Department of Biochemistry and Pharmacology, University of Melbourne, Parkville, Victoria 3052, Australia. e-mail: pcrack@unimelb.edu.au.

Acknowledgments:
The authors would like to thank A/Prof Eric Hanssen for assistance with the Transmission Electron Microscopy images and the Biological Optical Microscopy Platform (BOMP) with the Confocal Immunofluorescence microscopy. The authors would also like to thank the Biomedical Science Animal Facility (BSAF) at the University of Melbourne.
Peter J. Crack and Juliet M. Taylor are co-senior authors contributed equally to this work.

Authors' Contributions:
Harrison Waters and Shuyan Chen: Performed experiments. Elizabeth Vincan, Dustin J. Flanagan, Renate H.M. Schwab, Peter J. Crack, and Juliet M. Taylor: Provided experimental training. Harrison Waters, Peter J. Crack, and Juliet M. Taylor: Wrote the manuscript.

Conflicts of Interest:
The authors disclose no conflicts.

Funding:
The authors report no funding.

Ethical Statement:
All experimental procedures were conducted in compliance with the guidelines of the National Health and Medical Research Council (NHMRC) of Australia for animal experimentation. All procedures using mice were approved by the Animal Ethics Committee of the University of Melbourne (Ethics #22740).

Data Transparency Statement:
The authors declare that all data supporting the findings of this study are available within the paper and its Supplementary Material or from the corresponding authors on reasonable request.

Reporting Guidelines:
All experimental work involving animals followed the ARRIVE guidelines for animal research.

Gastro Hep Advances, Volume 5

Supplemental information

Type I Interferons Drive the Gastrointestinal Inflammatory Response in a Mouse Model of Parkinson's Disease

Harrison Waters, Shuyan Chen, Elizabeth Vincan, Dustin J. Flanagan, Renate H.M. Schwab, Peter J. Crack, and Juliet M. Taylor

Supplementary Methods

Cardiac puncture, plasma extraction, and tissue collection

WT and IFNAR1$^{-/-}$ mice were anesthetized via an i.p injection of Ketamine (100mg/kg) (ilium Ketamil) + Xylazine (10mg/kg) (ilium Xylazil-20). Blood was removed via cardiac puncture and plasma extracted. The colon was removed then the intestine was excised and separated into duodenum, jejunum and ileum with all tissues snap frozen in liquid nitrogen and stored at -80°C.

RNA isolation and cDNA synthesis

RNA was isolated from intestinal tissues (approximately 50-100mg) using TRIzol (Invitrogen, 15596018). Organoid samples were harvested using Corning® Cell Recovery Solution (Corning®, 354253) and pelleted before using RNAeasy mini kit (Qiagen, 74104) as per manufacturer's instructions. RNA samples were DNAse treated with an Ambion TURBO DNA-free™ Kit (Life Technologies), as per manufacturers guidelines. RNA was reverse transcribed into cDNA using a High-capacity RNA-to-cDNA Reverse Transcription Kit (4368814, Applied Biosystems)

Real time quantitative polymerase chain reaction (RT-qPCR)

All qPCR was performed in triplicate in standard 384-well plates (4309849, Applied Biosciences, Scoresby, VIC, Australia), with real time quantitative gene expression determined using Taqman probles (**Supp Table 2**) and analysed by the Quant Studio 6 Flex Real-Time PCR System (Invitrogen).

Western blot analysis

Western blot analysis was performed on 50µg of protein. Membranes were incubated with the primary antibodies **(Supp Table 3)** for 24 hours at 4°C and secondary antibodies for 1.5 hours at RT. Signals detected using an ECL Prime Detection kit (GE Healthcare Life Sciences) and visualised with a ChemiDoc™ imaging XRS+ system (Bio-Rad).

Enzyme Linked ImmunoSorbent Assay (ELISA)

A Mouse TNF-alpha Duoset ELISA (R&D Systems, DY410) was used to analyse TNFα levels in mouse plasma and in protein samples extracted from WT and IFNAR1$^{-/-}$ mice gut tissues.

Supplementary Tables

Cell Type	Antibody	Supplier	Primary dilution	Secondary Antibody
**All**	Rabbit Anti-alpha-synuclein	Cell Signalling (#4179)	1:200	Alexa Fluor® 488 Goat anti-mouse (A-11001)
**All**	Rabbit Phospho Anti-alpha-synuclein	Cell Signalling (#23706)	1:200	Alexa Fluor® 594 Goat anti-rabbit (A-21207)
**Organoids**	Purified Mouse Anti-E-cadherin monoclonal	BD Transduction Laboratories ™ (#610181)	1:300	Alexa Fluor® 594 Goat anti-rabbit (A-21207)
**Organoids**	Rabbit Lysozyme/Muramidase polyclonal	Thermo Scientific (RB-372)	1:200	Alexa Fluor® 488 Goat anti-mouse (A-11001)
**Organoids**	Mouse Chromogranin A monoclonal	Santa Cruz (#393941)	1:250	Alexa Fluor® 594 Goat anti-rabbit (A-21207)
**Whole gut IHC**	Mouse GFAP monoclonal	Cell Signalling (#3670)	1:1000	Alexa Fluor® 488 Goat anti-mouse (A-11001)

Table 1. Antibodies for immunofluorescence analysis

Gene	Species	Inventory #	Amplicon length (bp)
B2M	Mouse	Mm00437762_m1	77
IFNβ	Mouse	Mm00439552_s1	69
IRF7	Mouse	Mm00516788_m1	67
IL-1β	Mouse	Mm01336189_m1	63
TNFα	Mouse	Mm00443258_m1	81
IL-6	Mouse	Mm00446190_m1	78

Table 2. TaqMan probes used for qPCR analysis

Antibody	Type	Supplier	Catalogue #	Dilution	Species
**Anti-STAT1**	Primary	Cell Signalling	9172	1:1000	**Rabbit**
**Anti-Phospho-STAT1**	Primary	Cell Signalling	9167	1:1000	**Rabbit**
**Anti-NFκB p65**	Primary	Cell Signalling	8242	1:1000	**Rabbit**
**Anti-Phospho-NFκB p65**	Primary	Cell Signalling	3031	1:1000	**Rabbit**
**Anti-β-Actin**	Primary	Sigma-Aldrich	A5441	1:1000	**Mouse**
**Anti-GFAP**	Primary	Cell Signalling	3670S	1:1000	**Rabbit**
**Anti-IRF3**	Primary	Cell Signalling	4302S	1:1000	**Rabbit**
**Anti-STAT3**	Primary	Cell Signalling	4904	1:1000	**Rabbit**
**Anti-Phospho-STAT3**	Primary	Cell Signalling	9145S	1:1000	**Rabbit**
**Anti-IFNβ**	Primary	Santa Cruz	57201	1:500	**Rat**
**Anti-P-S129A αSynuclein**	Primary	Cell Signalling	23706	1:1000	**Rabbit**
**Anti-Total αSynuclein**	Primary	Cell Signalling	4179	1:1000	**Rabbit**
**Goat Anti-Rabbit Immunoglobulins/HRP**	Secondary	Dako	P0448	1:1000	**Goat**
**Goat Anti-Mouse Immunoglobulins/HRP**	Secondary	Dako	P0447	1:1000	**Goat**
**Goat Anti-Rat Immunoglobulins/HRP**	Secondary	Abcam	97057	1:1000	**Goat**

Table 3. Western blot analysis antibodies

Supplementary figures

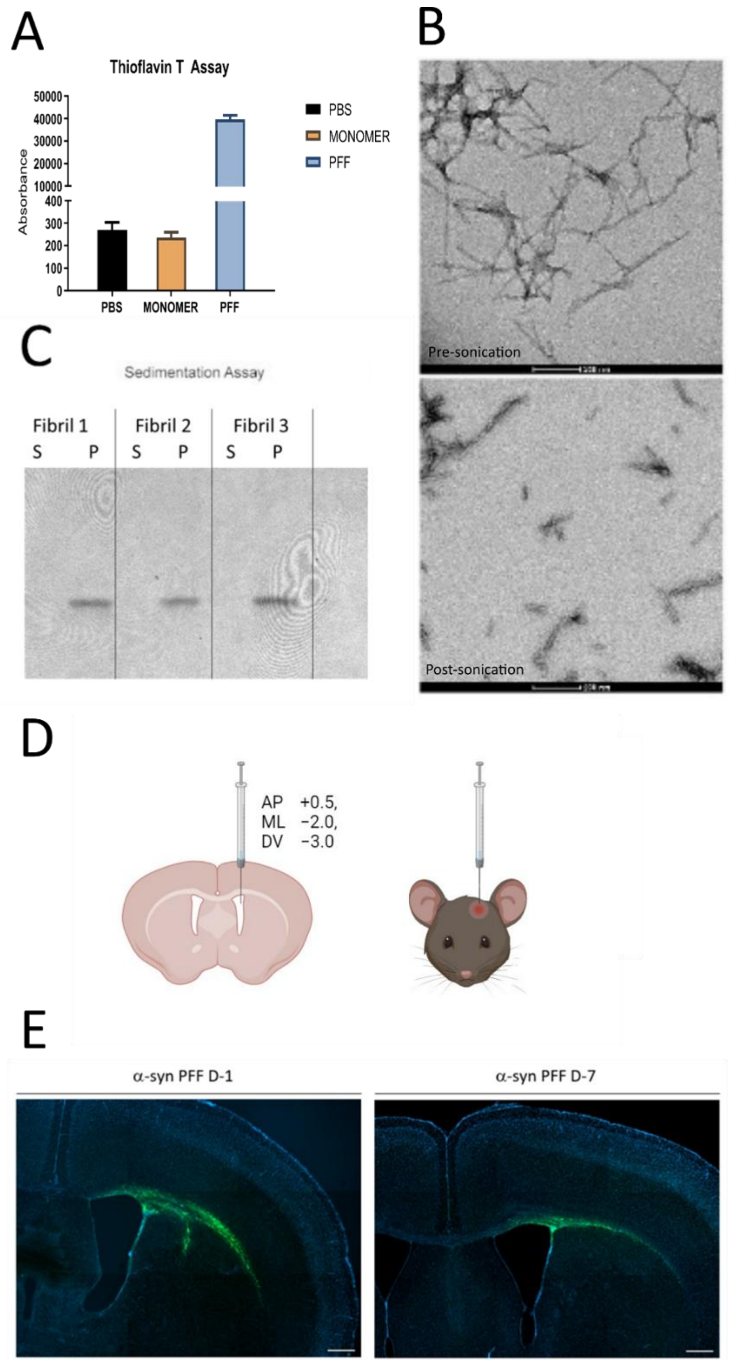

Supplementary Figure 1. Validation of the alpha synuclein PFF intrastriatal injection model

A, Thioflavin T assay confirming increased β-sheet structures in a-Synuclein PFF, when compared to both monomer and vehicle control. **B,** TEM images of pre- (i) and post- (ii) sonicated preparations of α-Syn PFFs showing decreased length (approximately 50nm) of fibrils. **C,** Sedimentation assay confirming the presence of α-Syn PFFs in the pelleted Representative images of 30µm cryosections of mouse brain at 1- and 7-days post injection with 488-labelled α-Syn PFFs. Co-stained with DAPI. Scale bar =100µm.

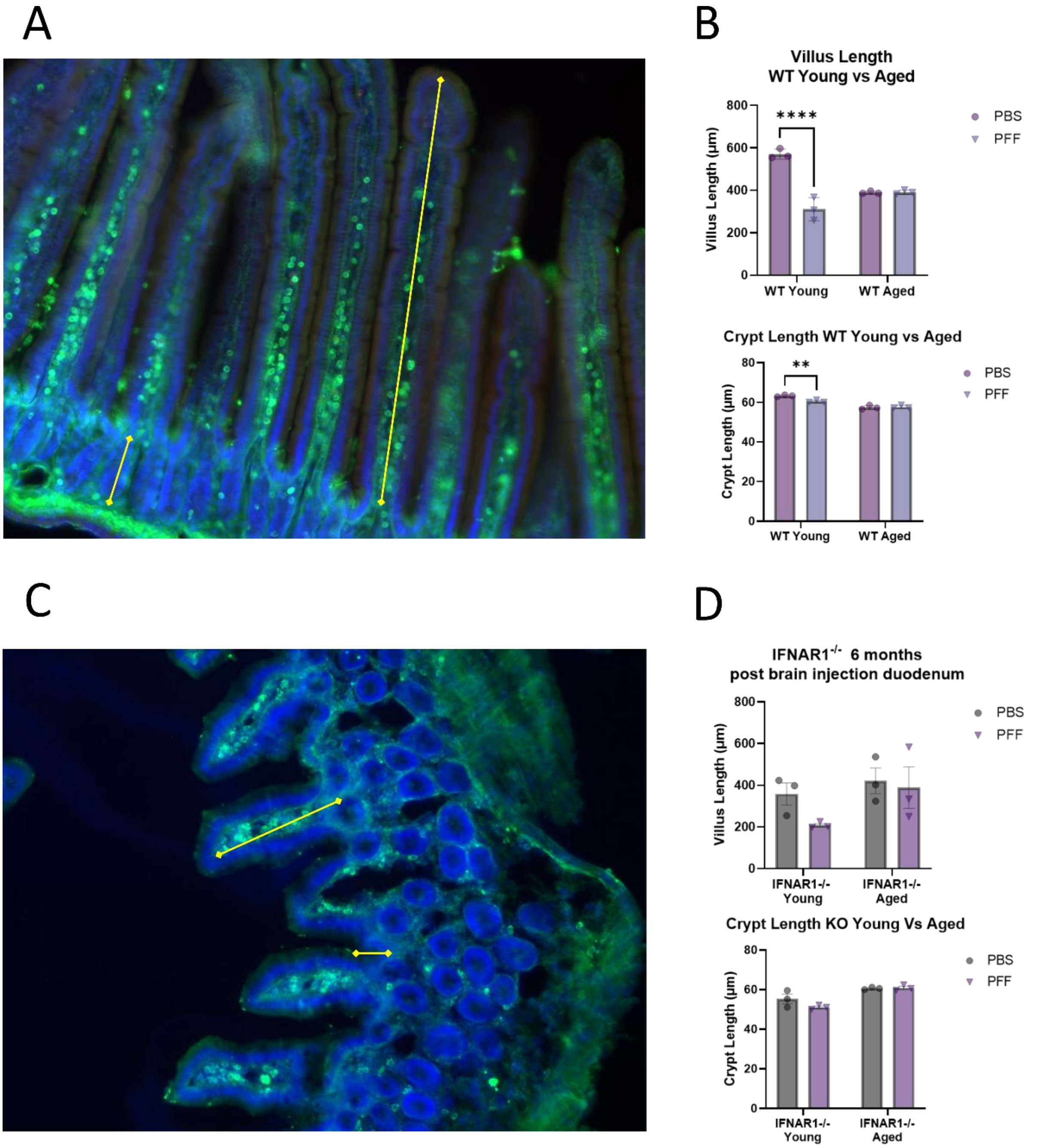

Supplementary Figure 2. Crypt and villus length is altered in young wildtype, but not IFNAR1$^{-/-}$ mice, following an intrastriatal injection of a-Syn PFFs (6-months p.i).

Young and aged WT **(A, B)** and IFNAR1$^{-/-}$ **(C, D)** mice duodenal tissue was analysed for villus and crypt length at 6 months post-injection of a-Syn PFFs or vehicle into the striatum. Representative images of 30μm cryosections stained with DAPI (blue) and Anti-GFAP (green) with 3 villus/crypt per cryosection counted, 10 cryosections per mouse, and 3 mice analysed. Data analysed with Two-way ANOVA, Tukey's Multiple Comparison's, data expressed as mean±SEM, n=3, **p ≤ 0.01, ****p≤ 0.0001.

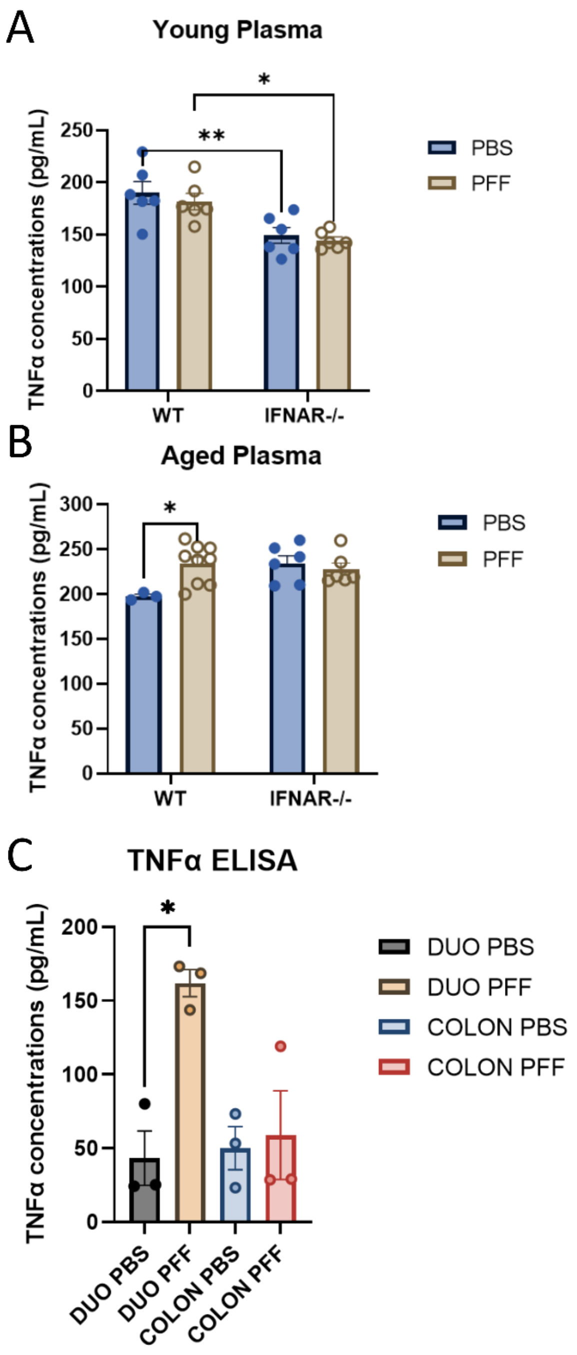

Supplementary Figure 3. TNFα levels are elevated in the plasma and duodenum of aged wildtype mice, but not IFNAR1$^{-/-}$ mice, following an intrastriatal injection of a-Syn PFFs (6-months p.i.)

Young (10-12 weeks of age) or Aged (40-50 weeks of age) wildtype and IFNAR1$^{-/-}$ animals were injected with a-Syn PFFs or vehicle and levels of TNFα in plasma (A, B) or duodenum and colon (C) determined by ELISA at 6-months post injection. Data expressed as mean±SEM, two-way ANOVA, Tukey's multiple comparisons test, * = p≤0.05, ** = p≤0.01.

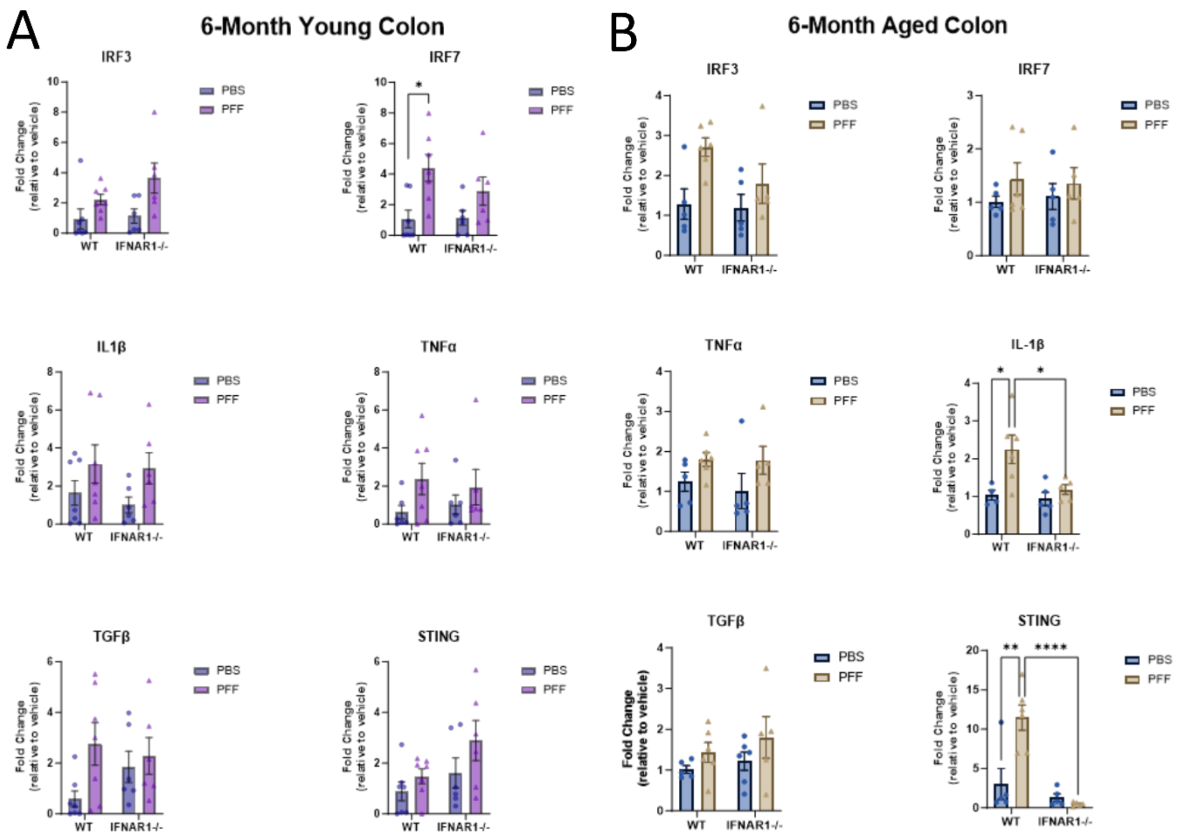

Supplementary Figure 4. IL1β and STING expression are upregulated in the colon of aged wildtype, but not IFNAR1$^{-/-}$ mice following an intrastriatal injection of a-Syn PFFs (6-months p.i.)

Colons from young (10-12 weeks of age when injected) **(A)** and aged (40-50 weeks of age when injected) **(B)** WT and IFNAR1$^{-/-}$ mice were analysed at 6-months after receiving an intrastriatal injection of α-Syn PFFs (8μg) or vehicle. mRNA expression of Interferon regulatory factor 3 & 7 (IRF3/7), Tumour Necrosis Factor Alpha (TNFα), Interleukin 1-beta (IL-1β), Transforming Growth Factor Beta (TGFβ), and Stimulator of Interferon Genes (STING) was determined by qPCR analysis. Data expressed relative to the housekeeping gene B2M and as fold change relative to vehicle control, mean±SEM, n=6-10, two-way ANOVA, Sidak's multiple comparison's test, *p≤0.05, **p≤0.01, ****p≤0.0001.
